# Global trends and regional disparities in the burden of headache disorders, 1990–2021: a comprehensive analysis of the global burden of disease study

**DOI:** 10.3389/fneur.2025.1575705

**Published:** 2025-06-05

**Authors:** Yuanyuan Rui, Bing Wu, Qian Li, Kai Zhang

**Affiliations:** ^1^Department of Emergency, The First Affiliated Hospital of Wannan Medical College, Wuhu, Anhui, China; ^2^Department of Emergency, The Second People’s Hospital of Lu’an City, Luan, Anhui, China; ^3^Department of Emergency, Xinhua Hospital Affiliated to Anhui University of Science and Technology, Huainan, Anhui, China

**Keywords:** headache disorders, global burden of disease, estimated annual percentage change, socio-demographic index, decomposition analysis, health inequality

## Abstract

**Background:**

Headache disorders significantly impact health and functioning, yet studies on their global burden across all age groups are limited. This study utilizes data from the Global Burden of Disease (GBD) 2021 to investigate the global burden of headache disorders.

**Methods:**

This analysis draws on GBD 2021 data, covering 204 countries and territories. We investigated the prevalence, incidence, and DALYs for headache disorders from 1990 to 2021, calculating Estimated Annual Percentage Change (EAPC) to analyze temporal trends. Additionally, decomposition analysis was used to evaluate the contributions of aging, population growth, and epidemiological changes. The slope index of inequality and concentration index were employed to assess inequalities in disease burden.

**Results:**

From 1990 to 2021, the global prevalence of headache disorders increased significantly, reaching approximately 2.81 billion cases in 2021, a 57.16% increase from approximately 1.79 billion cases in 1990. During this period, the global Age-Standardized Prevalence Rate (ASPR) and Age-Standardized DALY Rate (ASDR) both showed gradual increases, while the Age-Standardized Incidence Rate (ASIR) remained stable, with EAPC values of 0.01, 0.04 and-0.0002, respectively. High Socio-demographic Index (SDI) regions exhibited the highest rates of ASPR, ASIR, and ASDR, whereas Middle SDI regions experienced the fastest growth, with EAPC values of 0.17, 0.17, and 0.18, respectively. The SDI exhibited significant positive correlations with the EAPC of ASPR (*R* = 0.18, *p* = 0.0093) and ASIR (*R* = 0.16, *p* = 0.027). Decomposition analysis identified population growth as the primary driver in regions with increasing disease burden. The slope index of inequality (SII) shows that disparities in ASPR, ASIR, and ASDR slightly increased from 7,648.13, 2,506.76, and 88.45 in 1990 to 7,851.55, 2,557.94, and 100.38 in 2021. In contrast, the concentration index (CI) in 2021 were 0.05, 0.05, and 0.04, showing no significant change from 1990.

**Conclusion:**

Headache disorders continue to impose a growing burden globally, with marked regional and socio-economic disparities. Addressing these trends requires targeted public health interventions, particularly in high-burden and low-resource settings.

## Introduction

Headache disorders are among the most common and disabling neurological conditions globally, significantly impacting quality of life and productivity. They represent a substantial public health burden. Globally, headache disorders affect approximately 40% of the population in 2021, highlighting a vast population affected by headaches and posing significant challenges to healthcare system (1). The long-term impact of headache disorders, such as an elevated risk of comorbid conditions and diminished quality of life, highlights the urgency of addressing this issue. A comprehensive understanding of the epidemiology of headache disorders is vital for implementing early detection, preventive measures, and effective management strategies. These approaches are crucial for mitigating the enduring burden of the disorder and enhancing the overall outcomes for affected populations.

In 2021, headache disorders continued to be a major contributor to global disability-adjusted life years (DALYs) (2). This disabling condition is marked by frequent bouts of moderate to intense headaches, commonly associated with a range of neurological and systemic manifestations. These can include increased sensitivity to light and sound, along with gastrointestinal symptoms like nausea and vomiting (3, 4). Multiple factors can trigger headache disorder attacks, with common triggers including stress, exhaustion, and specific dietary elements (5). Identifying and managing these triggers is crucial for effective prevention and management of headache disorder. Specific dietary elements, alongside stress and exhaustion, are among the most common headache disorder triggers. Headache disorders are frequently undiagnosed and undertreated, especially in low- and middle-income countries due to limited research, education, and clinical resources (6).

In the Global Burden of Disease (GBD) database, the category of headache disorders includes migraine and tension-type headache. Previous research on headache disorders has predominantly targeted specific populations or age groups, with limited exploration of the burden across all age groups (1, 7, 8). This study seeks to fill this critical research gap by providing a comprehensive analysis of headache disorder epidemiology using the most recent GBD 2021 dataset. Advanced statistical techniques, such as Estimated Annual Percentage Change (EAPC), decomposition analysis, and assessments of health inequality, were employed to offer deeper insights into temporal trends and regional variations.

## Methods

### Data acquisition and download

The headache disorder data used in this study were sourced from the GBD 2021, which offers the most recent estimates of the burden from 371 diseases and injuries across 21 regions and 204 countries and territories from 1990 to 2021. These data are publicly available through the Global Health Data Exchange.^1^ Comprehensive details regarding data sources, methodologies, and statistical models are provided in earlier GBD reports. The GBD 2021 categorizes causes into four levels, from Level 1 (including communicable, maternal, neonatal, and nutritional diseases) to Level 4 (more specific conditions). In the GBD 2021, headache disorders are categorized as a Level 3 cause, positioning them within a specific classification in the broader framework of disease burden. This regional classification system, consistently used in prior GBD iterations, has proven to be a robust method for analyzing and comparing health metrics across diverse geographical and epidemiological contexts (9). The design of this system enables in-depth analysis of regional health disparities and trends in disease burden. For this study, data specific to headache disorders were extracted, focusing on prevalence, incidence, and DALYs. Each of these metrics was accompanied by their respective 95% uncertainty intervals (UI), providing a measure of statistical confidence and ensuring the robustness of the estimates.

### Disease definition

In the GBD 2021 framework, headache disorders are classified at Level 3, situated within the broader category of neurological disorders (Level 2), which is further encompassed by non-communicable diseases (Level 1). In GBD 2021, headache disorders include two conditions: migraine and tension-type headache. The diagnoses adhered to the International Classification of Diseases (ICD) codes, specifically ICD-10 G43 for migraines and G44 for tension-type headaches (2).

### Statistical analysis

To examine trends in age-standardized rates (ASR) of headache disorder prevalence, incidence, and DALYs, we applied the EAPC method. This method utilizes a regression model, which is formulated as (10):


ln(ASR)=α+β·(year)

Where β represents the annual change in the natural logarithm of ASR. The EAPC is then:


EAPC=100×(exp(β)−1).

An EAPC value is considered significant if its 95% confidence interval (CI) excludes zero.

Decomposition analysis was conducted to break down the observed changes in prevalence, incidence, and DALYs, attributing them to factors such as population growth, aging, and shifts in epidemiological trends (11, 12). Regional disparities were assessed using concentration indices and slope indices of inequality to evaluate variations in burden across socio-demographic index (SDI) quintiles (13). Statistical significance for comparative analyses was set at *p* < 0.05. All analyses were conducted using R software (version 4.1.2) visualizations.

## Results

### Global level

Globally, the prevalence, incidence, and DALYs of headache disorders have increased significantly over the past three decades (Table 1; Supplementary Tables S1, S2). From 1990 to 2021, the estimated number of prevalent cases rose by 57.16%, from 1,787,302,945.09 cases (95% UI: 1,648,822,201.72–1,937,346,555.85) in 1990 to 2,808,876,481.80 cases (95% UI: 2,599,555,367.51–3,028,767,343.34) in 2021, while the global Age-Standardized Prevalence Rate (ASPR) increased slightly from 34,486.61 to 34,574 per 100,000 population. Incident cases also showed a marked rise of 51.6%, increasing from 110,194,635.29 cases (95% UI: 47,246,499.69–59,143,093.16) in 1990 to 809,226,480.19 cases (95% UI: 717,818,771.04–895,990,201.46) in 2021. Similarly, DALYs attributable to headache disorders surged by 58.54%, from 30,260,883.92 (95% UI: 5,963,391.97–64,833,434.46) in 1990 to 47,975,675.06 (95% UI: 9,800,212.26–100,667,852.52) in 2021. These findings underscore the substantial and growing burden of headache disorders on global health.

**Table 1:** Prevalence of headache disorder between 1990 and 2021 at the global and regional level.

Location	1990		2021		1990–2021	
	Number (95%UI)	ASR (95%UI)	Number (95%UI)	ASR (95%UI)	Cases change (95%UI)	EAPC_95%CI
Global	1787302945.09 (1648822201.72, 1937346555.85)	34486.61 (31872.81, 37187.63)	2808876481.80 (2599555367.51, 3028767343.34)	34574.00 (32017.69, 37318.55)	57.16 (54.70, 59.74)	0.01 (−0.00, 0.03)
High SDI	377303232.74 (348181382.12, 406006717.17)	40362.19 (37304.58, 43443.77)	469853685.52 (432991273.76, 502490636.58)	39943.22(36968.04, 42992.91)	24.53 (22.11, 26.92)	−0.04(−0.05, −0.02)
High-middle SDI	364326745.53 (335902461.66, 394839881.48)	33201.02 (30676.58, 35848.97)	470255760.66 (434045840.95, 507003958.29)	33265.16 (30732.39, 35977.83)	29.08 (25.95, 32.57)	0.03 (0.00, 0.05)
Middle SDI	535062611.34 (491821074.37, 580740601.30)	31883.97 (29499.33, 34375.46)	862941418.52 (798706924.00, 930877361.47)	33529.25 (31076.45, 36180.36)	61.28 (56.87, 66.05)	0.17 (0.15, 0.19)
Low-middle SDI	372562916.41 (340616160.71, 405930156.02)	35304.29 (32608.81, 38179.70)	681846992.37 (628437764.17, 739801197.11)	35291.68 (32601.54, 38134.86)	83.02 (79.86, 86.00)	−0.01(−0.03, 0.00)
Low SDI	136234425.30 (123294936.12, 149672772.29)	32130.34 (29507.18, 34967.66)	321669319.71 (291652121.61, 353731234.08)	31917.54 (29344.65, 34700.57)	136.11 (134.76, 137.40)	−0.03(−0.04, −0.02)
Andean Latin America	10061024.49 (9099095.08, 11152453.96)	27981.28 (25592.45, 30603.19)	19322851.74 (17463982.69, 21373104.85)	28686.66 (25949.90, 31650.66)	92.06 (85.19, 99.14)	0.10 (0.08, 0.12)
Australasia	7695930.81 (7049006.39, 8401347.27)	36020.01 (33048.09, 39251.67)	11825960.45 (10804070.81, 12817469.18)	36018.00 (33015.95,39258.62)	53.67 (50.91, 56.40)	0.00(−0.00, 0.00)
Caribbean	11780082.00 (10615140.31, 13020833.61)	33902.81 (30873.00, 37146.67)	16599788.36 (15142153.49, 18174700.54)	33869.58 (30851.11, 37119.95)	40.91 (37.75, 44.32)	−0.00(−0.00, −0.00)
Central Asia	25272686.37 (22863066.12, 27916958.98)	38846.10 (35330.90, 42506.65)	37359630.53 (34003364.82, 40989853.88)	38788.18 (35324.88, 42457.39)	47.83 (44.56, 51.12)	−0.01(−0.01, −0.00)
Central Europe	51248153.32 (47135195.79, 55620935.90)	39086.17 (35859.50, 42620.91)	48986031.61 (44773980.88, 52892512.71)	39073.11 (35876.92,42546.93)	−4.41(−6.63, −2.39)	−0.00(−0.01, −0.00)
Central Latin America	52807613.77 (48056214.58, 57997064.28)	34241.46 (31563.94, 37264.51)	90477318.26 (83266977.22, 98500172.96)	34380.69 (31661.59, 37419.98)	71.33 (66.62, 76.54)	0.02 (0.01, 0.02)
Central Sub-Saharan Africa	14774665.29 (13239356.29, 16569474.73)	32086.76 (29166.59, 35428.08)	38908597.84 (34926922.11, 43572462.39)	32061.77 (29150.09, 35404.67)	163.35 (162.09, 164.78)	−0.00(−0.00, −0.00)
East Asia	320533274.09 (293466636.51, 349968249.84)	25844.47 (23775.25, 27983.66)	441138886.87 (406671695.43, 478351948.82)	27569.59 (25452.72, 29984.12)	37.63 (31.66, 44.22)	0.24 (0.19, 0.29)
Eastern Europe	94586699.93 (87424520.18, 102207997.14)	39806.68 (36827.38, 42995.07)	89285285.39 (82617768.80, 95996350.07)	39866.60 (36979.81,43073.94)	−5.60(−7.49,-3.71)	0.01 (0.00, 0.01)
Eastern Sub-Saharan Africa	41001831.52 (36531463.93, 45726019.12)	26023.58 (23629.89, 28766.78)	98354293.61 (87960669.29, 109430326.24)	25769.42 (23341.19, 28330.79)	139.88 (137.56, 141.91)	−0.05(−0.06, −0.03)
High-income Asia Pacific	68320083.50 (62899636.74, 74076924.25)	36502.70 (33628.94, 39715.32)	74015049.85 (67774802.68, 79837896.30)	36759.15 (33826.67, 40033.30)	8.34 (4.35, 12.63)	0.05 (0.04, 0.07)
High-income North America	131961655.36 (122553855.49, 141962034.62)	44486.85 (41339.93, 47815.49)	171535442.63 (159141040.49, 183173917.40)	43612.53 (40628.86, 46915.07)	29.99 (26.91, 33.08)	−0.07(−0.10, −0.04)
North Africa and Middle East	106319396.65 (96056204.34, 117105956.39)	34767.16 (31990.50, 37850.97)	219957170.86 (201320362.82, 239186127.41)	34984.20 (32119.27, 37886.31)	106.88 (101.75, 112.27)	0.03 (0.01, 0.04)
Oceania	1909134.29 (1705425.03, 2098227.35)	32342.59 (29243.36, 35334.12)	4235347.35 (3809401.96, 4661433.19)	32361.94 (29271.16, 35375.72)	121.85 (119.12, 124.67)	0.00 (0.00, 0.00)
South Asia	358358212.23 (328459852.61, 390526009.20)	35635.64 (32914.07, 38524.34)	680674142.02 (628003483.67, 736856961.56)	35688.00 (32985.80, 38470.02)	89.94 (86.46, 93.63)	−0.02(−0.04, 0.01)
Southeast Asia	161277340.49 (147632407.52, 174994436.29)	36609.57 (33825.23, 39563.12)	265769743.15 (245290679.14, 287027698.98)	36398.51 (33614.82, 39330.25)	64.79 (60.69, 69.10)	−0.02(−0.02, −0.02)
Southern Latin America	16536928.25 (15013697.08, 18177406.04)	33682.48 (30616.33, 36968.62)	24445106.86 (22192453.03, 26859083.83)	34056.88 (30849.24, 37424.65)	47.82 (44.89, 50.74)	0.05 (0.04, 0.06)
Southern Sub-Saharan Africa	15887310.65 (14497125.77, 17402985.36)	33225.40 (30568.92, 36062.25)	26906114.27 (24714304.49, 29384899.42)	33198.87 (30538.15, 36048.13)	69.36 (66.08, 72.66)	−0.00(−0.00, −0.00)
Tropical Latin America	61591490.39 (56830235.76, 66692177.95)	40772.19 (37721.11, 43816.40)	97370535.40 (90317007.00, 105222983.21)	41148.76 (38049.51, 44522.12)	58.09 (53.15, 63.25)	0.01(−0.01,0.02)
Western Europe	178016065.07 (164119893.10, 191571179.80)	43249.49 (39887.15, 46586.01)	201163898.69 (185551318.08, 216210145.68)	43409.83 (40111.93, 46783.53)	13.00 (11.05, 14.93)	0.04 (0.03, 0.05)
Western Sub-Saharan Africa	57363366.62 (52136137.46, 62731868.41)	35301.18 (32414.09, 38234.47)	150545286.06 (136632772.52, 165121461.07)	35346.21 (32436.38, 38342.04)	162.44(160.45, 164.21)	−0.00(−0.01, 0.01)

EAPC, estimated annual percentage change; SDI, sociodemographic Index; UI, uncertainty interval; CI, confidence interval; ASR, age-standardized rates.

**Figure 1:**
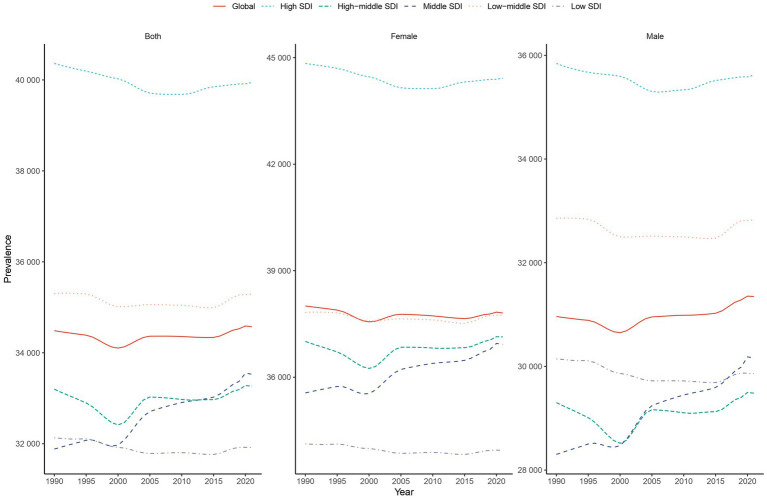
Trends of prevalence in headache disorder from 1990 to 2021. The global ASPR of headache disorders from 1990 to 2021, disaggregated by SDI regions and sex. **(A)** Represents data for both sexes combined, **(B)** for females, and **(C)** for males. High SDI regions consistently exhibit the highest ASPR across all years, while low SDI regions report the lowest rates. Trends highlight a gradual increase in ASPR for low- and middle-SDI regions, with minimal variation in high-SDI regions. The gap between male and female ASPR is evident across all SDI regions, with females consistently experiencing higher prevalence rates. SDI, Socio-demographic Index; ASPR, Age-Standardized Prevalence Rate.

From 1990 to 2021, the global ASPR and Age-Standardized DALYs Rate (ASDR) exhibited a gradual upward trend, while the Age-Standardized Incidence Rate (ASIR) remained relatively stable (Figure 1; Supplementary Figures S1, S2). The EAPC for ASPR, ASIR, and ASDR were 0.01 (95% UI: −0.00 to 0.03), −0.0002 (95% UI: −0.01 to 0.01), and 0.04 (95% UI: 0.03 to 0.05), respectively (Supplementary Figures S3–S5, Table 1; Supplementary Tables S1, S2). Although females consistently demonstrated higher ASPR, ASIR, and ASDR compared to males, the upward trend in these metrics was more pronounced among males. The ASPR in males increased from 30,962.89 per 100,000 population (95% UI: 28,383.19–33,621.16) in 1990 to 31,347.76 per 100,000 population (95% UI: 28,813.74–34,106.65) in 2021. Similarly, the ASIR in males rose from 9,461.78 per 100,000 population (95% UI: 8,356.71–10,529.63) in 1990 to 9,536.94 per 100,000 population (95% UI: 8,430.79–10,583.06) in 2021. Notably, the ASDR for males increased from 439.74 cases (95% UI: 921.84–99.90) per 100,000 population in 1990 to 453.22 cases (95% UI: 959.01–97.50) per 100,000 population in 2021.

### SDI regional level

In 2021, the Middle SDI region reported the highest number of cases and emerged as the epicenter of the headache disorder burden. The number of prevalent cases was 862,941,418.52 (95% UI: 798,706,924.00–930,877,361.47), while the number of incident cases reached 243,446,537.63 (95% UI: 215,909,105.51–269,427,846.80), and the DALYs totaled 15,284,069.03 (95% UI: 2,903,732.51–32,354,748.10) (Table 1; Supplementary Tables S1, S2). However, the ASPR, ASIR, and ASDR were highest in the High SDI region (Table 1; Supplementary Tables S1, S2). From 1990 to 2021, the High SDI region consistently exhibited the highest rates for these metrics (Figure 1; Supplementary Figures S1, S2). Notably, the Middle SDI region experienced the fastest growth in ASPR, ASIR, and ASDR, with corresponding EAPC values of 0.17 (95% UI: 0.15–0.19), 0.17 (95% UI: 0.16–0.19), and 0.18 (95% UI: 0.17–0.20), respectively (Supplementary Figures S3–S5; Table 1; Supplementary Tables S1, S2).

### GBD regional level

South Asia emerged as the epicenter of the prevalence burden, with an estimated 680,674,142.02 cases (95% UI: 628,003,483.67–736,856,961.56), while Oceania reported the lowest burden, with only 4,235,347.35 cases (95% UI: 3,809,401.96–4,661,433.19). Among the 21 GBD regions, 19 regions experienced increases in the number of prevalence cases, incidence cases, and DALYs. However, in some High and Middle SDI regions, such as Central Europe and Eastern Europe, these three metrics for headache disorders showed a declining trend (Table 1; Supplementary Tables S1, S2).

Over the past three decades, East Asia has shown the most significant increases in ASPR, ASIR, and ASDR, with EAPC values of 0.24 (95% UI: 0.19–0.29), 0.22 (95% UI: 0.17–0.26), and 0.25 (95% UI: 0.21–0.29), respectively. In contrast, High-Income North America experienced the steepest decline in ASPR (EAPC: -0.07; 95% UI: −0.10 to−0.04), while Eastern Sub-Saharan Africa showed the fastest decrease in ASIR (EAPC: −0.06; 95% UI: −0.08 to −0.05). In 2021, High-Income North America recorded the highest ASPR and ASIR globally, at 43,612.53 per 100,000 population (95% UI: 40,628.86–46,915.07) and 13,330.95 per 100,000 population (95% UI: 11,802.72–14,776.65), respectively. Conversely, Eastern Sub-Saharan Africa reported the lowest values for these metrics, with an ASPR of 25,769.42 per 100,000 population (95% UI: 23,341.19–28,330.79) and an ASIR of 7,797.13 per 100,000 population (95% UI: 6,869.75–8,703.11). Western Europe had the highest ASDR, at 748.58 per 100,000 population (95% UI: 144.83–1,578.77), whereas Eastern Sub-Saharan Africa had the lowest ASDR (Supplementary Figures S3–S5; Table 1; Supplementary Tables S1, S2).

**Figure 2:**
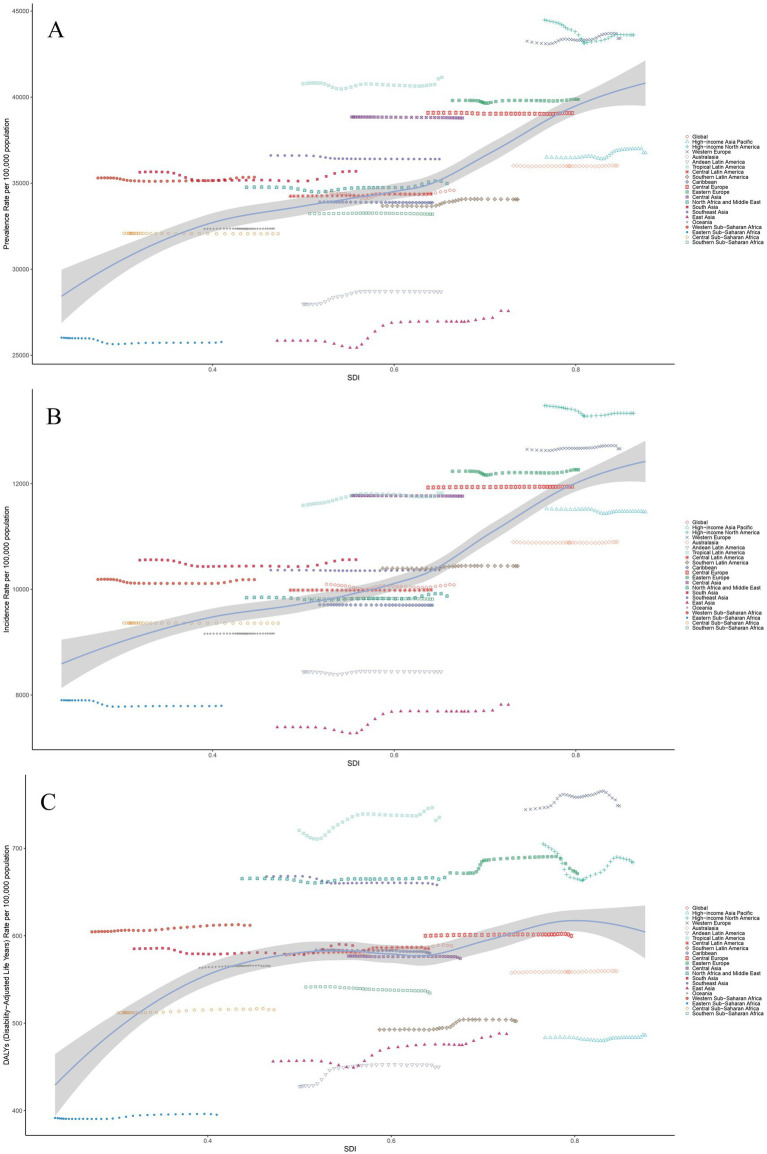
The associations between the SDI and headache disorder across 21 GBD regions. **(A)** Association between ASPR and SDI. **(B)** Association between ASIR and SDI. **(C)** Association between ASDR and SDI. **(A–C)** The association between SDI and ASPR, ASIR, and ASDR, respectively, across 21 GBD regions in 2021. The lines represent the overall trends, with individual points corresponding to each region, colored by their respective regions. Shaded areas indicate 95% uncertainty intervals. Notable variability is observed among regions, reflecting distinct socio-demographic and healthcare factors influencing the burden of headache disorders. ASPR, Age-Standardized Prevalence Rate; ASIR, Age-Standardized Incidence Rate; ASDR, Age-Standardized Disability-Adjusted Life Years; SDI, Socio-demographic Index; DALYs, Disability-Adjusted Life Years.

Figure 2 illustrates positive correlations between the ASPR (*r* = 0.58, *p* < 0.001), ASIR (*r* = 0.64, *p* < 0.001), and ASDR (*r* = 0.24, *p* < 0.001) of headache disorders and the SDI.

### Countries level

**Figure 3:**
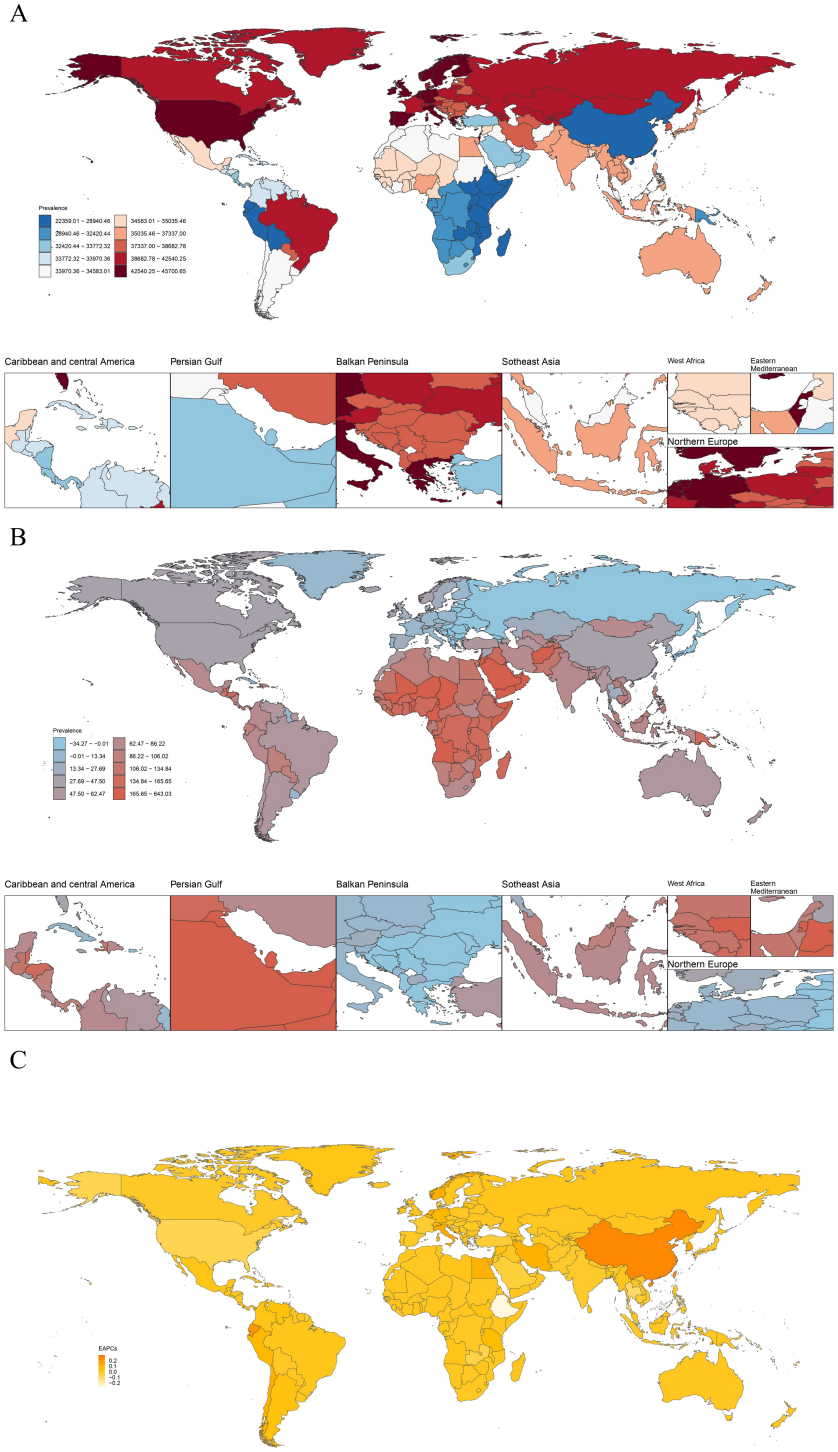
**(A)** The ASPR of headache disorders in 204 countries and territories in 2021. **(B)** The case changes in the prevalence of headache disorders across 204 countries and territories from 1990 to 2021. **(C)** EAPC of ASPR. Global ASPR of headache disorders per 100,000 population in 2021. Darker shades indicate higher prevalence, with detailed regional focus in Caribbean and Central America, Persian Gulf, Balkan Peninsula, Southeast Asia, West Africa, Eastern Mediterranean, and Northern Europe. Percent change in prevalence rates of headache disorders from 1990 to 2021, showing areas with significant increases or decreases. EAPCs of ASPR of headache disorder from 1990 to 2021, highlighting regions with notable growth or decline. ASPR, age-standardized prevalence rate; EAPC, estimated annual percentage change.

The global map of headache prevalence from 1990 to 2021 revealed substantial differences among countries. In 2021, among the 204 countries analyzed, Belgium recorded the highest ASPR of headache disorders, with 45,248.17 cases per 100,000 population (95% UI: 41,636.70–48,749.13), establishing itself as the epicenter of headache burden. In contrast, Ethiopia had the lowest ASPR, with 22,584.86 cases per 100,000 population (95% UI: 20,367.11–24,914.66) (Figure 3A). In terms of absolute case numbers, Qatar experienced the fastest increase, with a rise of 636%, followed by the United Arab Emirates at 492% (Figure 3B). Regarding ASPR trends, Singapore exhibited the most significant increase (EAPC: 0.29; 95% UI: 0.21–0.38), whereas Ethiopia showed the steepest decline in ASPR (EAPC: −0.26; 95% UI: −0.31 to −0.21) over the same period (Figure 3C).

In 2021, Norway recorded the highest ASIR of headache disorders, with 13,651.15 cases per 100,000 population (95% UI: 12,119.39–15,249.30) (Supplementary Figure S6A). In terms of the absolute number of new cases, Qatar showed the largest increase, with a rise of 608% (Supplementary Figure S6B). Regarding ASIR trends, China exhibited the fastest growth (EAPC: 0.22; 95% UI: 0.17–0.27), while Ethiopia experienced the steepest decline (EAPC: −0.29; 95% UI: −0.34 to −0.23) (Supplementary Figure S6C). For DALYs, Belgium had the highest ASDR in 2021, at 869.77 per 100,000 population (95% UI: 135.02–1,852.24) (Supplementary Figure S7A). Qatar also saw the largest increase in absolute DALYs, rising by 638% (Supplementary Figure S7B). Regarding ASDR trends, Singapore showed the most significant increase (EAPC: 0.36; 95% UI: 0.26–0.46), while Thailand experienced the sharpest decline (EAPC: −0.27; 95% UI: −0.35 to −0.18) (Supplementary Figure S7C). These results emphasize significant regional variations in the burden of headache disorders and highlight countries with the fastest-growing or declining trends in incidence and disability.

**Figure 4:**
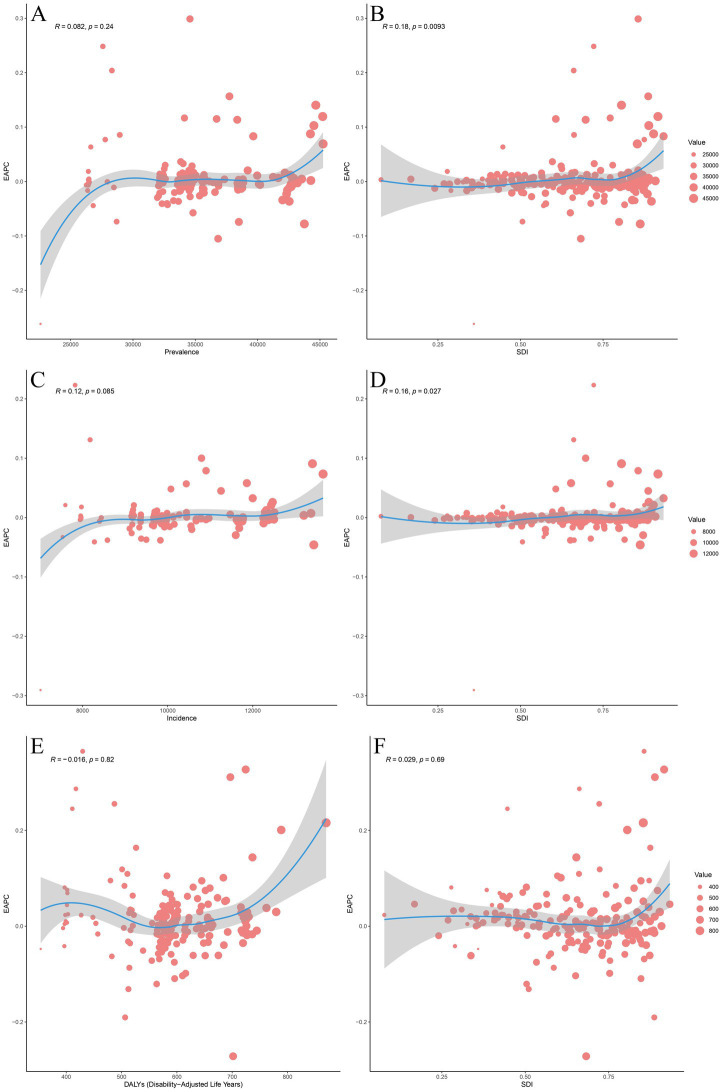
The associations between EAPC and ASPR, ASIR, ASDR, and SDI. **(A)** The association between EAPC and ASPR in 2021. **(B)** The association between EAPC of ASPR and SDI. **(C)** The association between EAPC and ASIR in 2021. **(D)** The association between EAPC of ASIR and SDI in 2021. **(E)** The association between EAPC and ASDR in 2021. **(F)** The association between EAPC of ASDR and SDI. The association between EAPC of ASPR and ASPR for headache disorder across 204 countries and territories in 2021. The size of the circle increases with the number of prevalence. The R indices and *p* values were derived from Pearson correlation analysis. The association between EAPC of ASPR and SDI for headache disorder across 204 countries and territories in 2021. The blue line represents expected values based on SDI and disease across 204 countries and territories; each point shows the observed ASPR for the specified countries in 2021. The association between EAPC of ASIR and ASIR for headache disorder across 204 countries and territories in 2021. The size of the circle increases with the number of incidence. The R indices and *p* values were derived from Pearson correlation analysis. The association between EAPC of ASIR and SDI for headache disorder across 204 countries and territories in 2021. The blue line represents expected values based on SDI and disease across 204 countries and territories; each point shows the observed ASIR for the specified countries in 2021. The association between EAPC of ASDR and ASDR for headache disorder across 204 countries and territories in 2021. The size of the circle increases with the number of DALYs. The R indices and *p* values were derived from Pearson correlation analysis. The association between EAPC of ASDR and SDI for headache disorder across 204 countries and territories in 2021. The blue line represents expected values based on SDI and disease across 204 countries and territories; each point shows the observed ASDR for the specified countries in 2021. EAPC, Estimated Annual Percentage Change; ASPR, Age-Standardized Prevalence Rate; ASIR, Age-Standardized Incidence Rate; ASDR, Age-Standardized Disability-Adjusted Life Years; SDI, Socio-demographic Index; DALYs, Disability-Adjusted Life Years.

Supplementary Figure S8 illustrates the positive correlations between the ASPR (*r* = 0.52, *p* < 0.001), ASIR (*r* = 0.53, *p* < 0.001), and ASDR (*r* = 0.39, *p* < 0.001) of headache disorders with the SDI across 204 countries and regions. EAPC showed no significant correlation with the ASPR (*R* = 0.082, *p* = 0.24) and ASIR (*R* = 0.12, *p* = 0.085) across 204 countries in 2021 (Figures 4A,C). In contrast, the SDI exhibited significant positive correlations with the EAPC of ASPR (*R* = 0.18, *p* = 0.009) and ASIR (*R* = 0.16, *p* = 0.027) (Figure 4B; Figure 4D). Regarding DALYs, no significant correlations were observed between EAPC and DALYs (*R* = −0.016, *p* = 0.82) or between SDI and the EAPC of ASDR (*R* = 0.029, *p* = 0.69) (Figures 4E,F).

### Age and sex patterns

**Figure 5:**
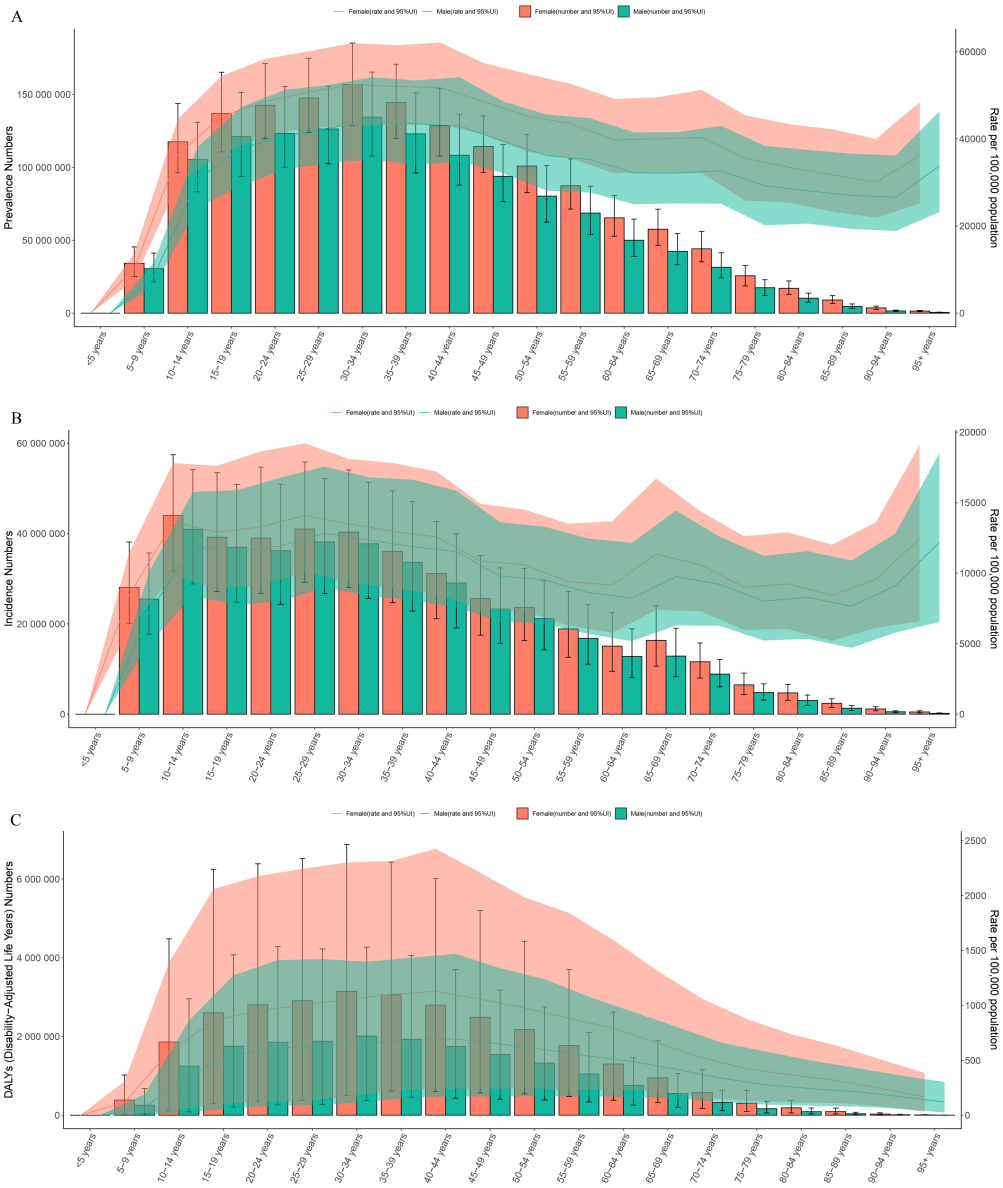
The bar charts represent the number of prevalence, incidence, and DALYs across different age groups, while the line charts show the prevalence, incidence, and DALY rates. **(A)** Prevalence. **(B)** Incidence. **(C)** DALYs. Prevalence by age group and gender, with male and female data presented as the number of cases and rates (per 100,000 population) in 2021. Error bars indicate the 95% uncertainty intervals. Female values are represented in red, and male values in green. Incidence by age group and gender in 2021, similar to **(A)**, displaying data as counts and rates. **(C)** DALYs by age group and gender, illustrating the burden across all age groups. The size of each bar is proportional to the burden represented by DALYs, with red indicating female and green indicating male, along with 95% UI. DALYs, Disability-Adjusted Life Years; UI, Uncertainty Interval.

In 2021, the global prevalence of headache disorders exhibited a marked increase in the 5–9 age group, peaking in the 30–34 age group. The prevalence rates for males and females aged 30–34 were 44,011.60 cases (95% UI: 35,272.57–54,126.32) per 100,000 population and 55,243.91 cases (95% UI: 43,023.90–61,984.53) per 100,000 population, respectively. The highest number of prevalent cases was observed in the 30–34 age group, which subsequently declined with advancing age. The number of prevalence for males and females aged 30–34 was 134,477,150.70 (95% UI: 107,775,101.48–165,382,616.34) and 156,756,114.79 (95% UI: 128,611,719.75–185,290,902.74), respectively. Across all age groups, females consistently had higher prevalence rates and numbers than males (Figure 5A). The incidence rate peaked in the 25–29 age group, then gradually declined, with an upward trend observed in the 95 + age group. The incidence rates for males and females aged 25–29 were 12,830.43 cases (95% UI: 8,980.81–17,560.10) and 14,092.50 cases (95% UI: 10,040.23–19,198.79), respectively. The highest number of incident cases was in the 10–14 age group, which decreased thereafter. The number of incidence for males and females aged 10–14 was 40,972,899.04 (95% UI: 28,844,584.83–54,167,271.74) and 44,012,691.59 (95% UI: 31,718,096.81–57,477,937.31), respectively. Similarly, females had higher incidence rates and case numbers across all age groups compared to males (Figure 5B). The DALY rate peaked in the 40–44 age group, while the total DALY count reached its highest in the 30–34 age group. The DALY rates for males and females aged 40–44 were 693.25 (95% UI: 171.37–1,469.54) and 1,127.94 (95% UI: 241.28–2,425.05), respectively. The number of DALYs for males and females aged 30–34 was 2,010,632.08 cases (95% UI: 372,659.61–4,270,425.88) and 3,147,997.45 cases (95% UI: 501,654.99–6,879,635.15), respectively. Across all age groups, females exhibited higher DALY rates and counts than males (Figure 5C).

### Decomposition analysis from 1990 to 2021 according to SDI and 21 GBD regions

**Figure 6:**
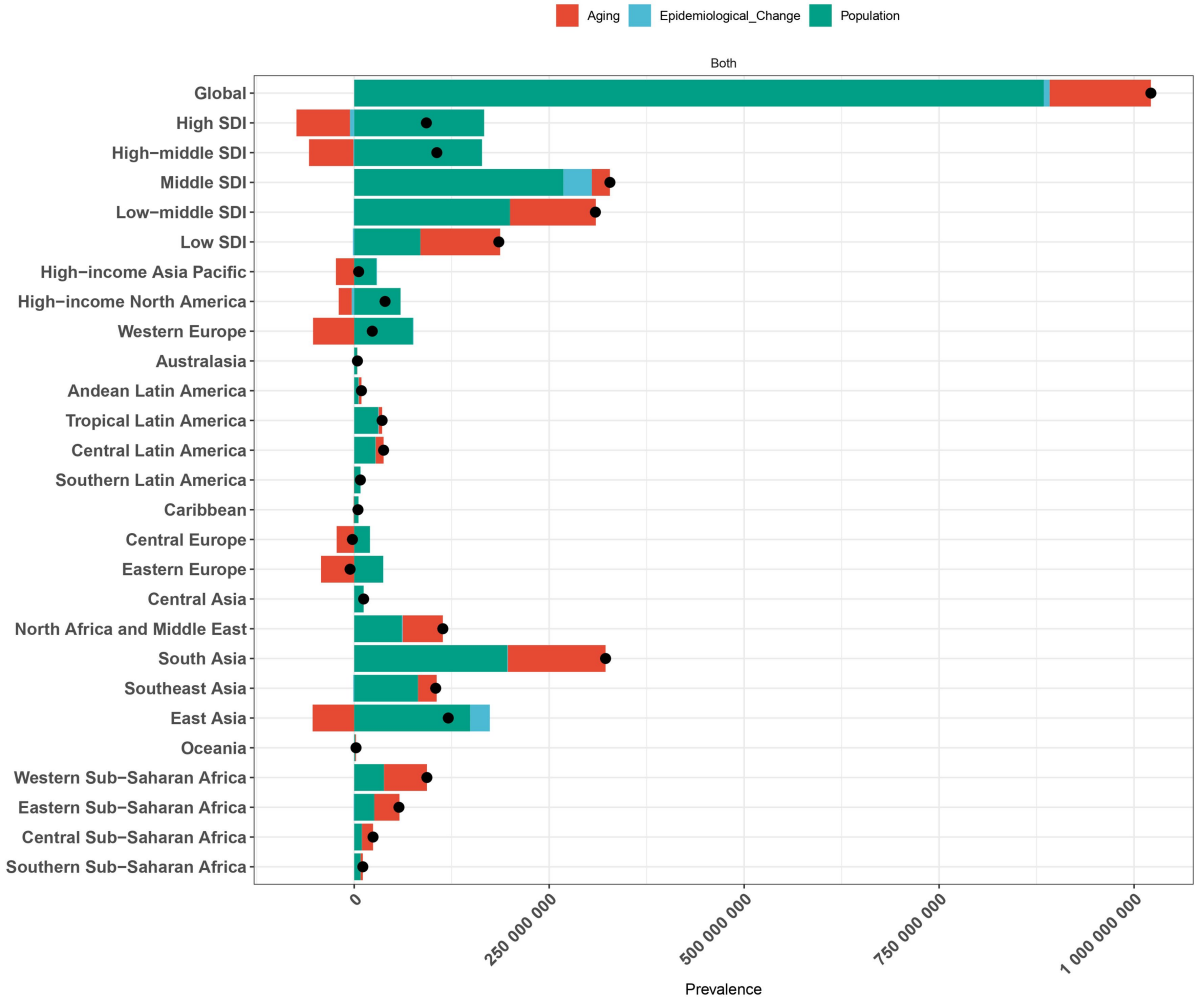
Decomposition analysis of headache disorder change in prevalence by SDI and 21 GBD regions, 1990 to 2021. Decomposition of the change in the prevalence of headache disorders from 1990 to 2021 by regions, showing contributions from aging, epidemiological change, and population growth. Red bars represent the contribution of aging, blue bars indicate epidemiological change, and green bars show population growth. The size of each bar corresponds to the respective regional contribution. The black dots represent the overall trend in disease burden change for each region. SDI, Sociodemographic Index; GBD, Global burden of disease.

Our detailed decomposition analysis highlighted the relative impacts of aging, population growth, and demographically adjusted epidemiological changes on the trends in headache disorder incidence, prevalence, and DALYs across the five SDI regions and 21 GBD regions. From 1990 to 2021, the global ASPR, ASIR, and ASDR for headache disorders increased significantly, with population growth identified as the primary contributor to the increased burden. The contributions of population growth to the increase in ASPR, ASIR, and ASDR were 884,321,596.70 (86.56%), 262,451,776.68 (95.29%), and 15,038,180 (84.89%), respectively. Across all five SDI regions, the burden of headache disorders also rose significantly. In low SDI regions, population aging emerged as the dominant factor, contributing 102,288,334.5 (55.16%), 31,076,851.66 (54.63%), and 1,676,519.06 (54.56%) to the increases in ASPR, ASIR, and ASDR, respectively. In contrast, in other SDI regions, population growth was the primary driver of the increased burden. Among the 21 GBD regions, the burden of headache disorders decreased in Central Europe and Eastern Europe, with population aging being the key factor driving this decline. In Central Europe, population aging contributed to reductions of-22,463,264.06 (993.02%), −7,068,799.17 (575.14%), and −343,726.53 (1210.52%) in ASPR, ASIR, and ASDR, respectively. Similarly, in Eastern Europe, aging contributed to decreases of −42,556,584.22 (802.74%), −13,304,457.51 (563.75%), and −706,738.61 (1146.54%) in ASPR, ASIR, and ASDR, respectively. In contrast, all other GBD regions experienced an increase in the burden, primarily driven by population growth in most regions (Figure 6; Supplementary Figures S9, S10).

### Cross-national health inequality

**Figure 7:**
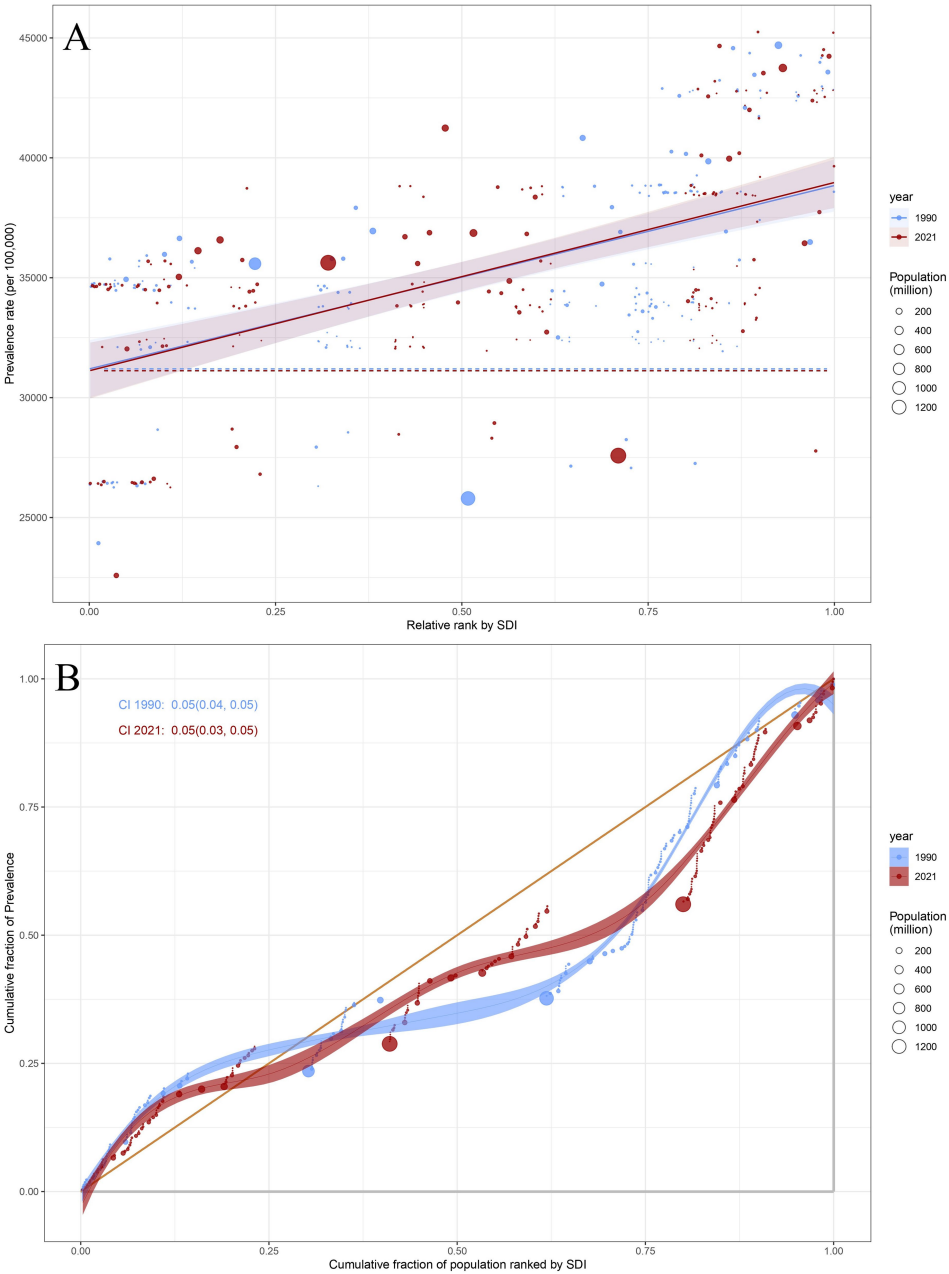
**(A)** Health inequality regression curves of prevalence for headache disorders. **(B)** Concentration curves of prevalence for headache disorders. **(A)** The relationship between ASPR and relative rank by SDI for headache disorders across 204 countries in 1990 and 2021. Points represent countries with varying population sizes, denoted by the size of the circle (population in millions). The blue dotted line represents the trend for 1990, and the red solid line shows the trend for 2021, along with shaded confidence intervals. **(B)** The cumulative fraction of ASPR for headache disorders across 204 countries in 1990 and 2021. Points represent the cumulative fraction of prevalence across different SDI categories, while the line reflects the cumulative trends in prevalence rates. The CI values for 1990 and 2021 are shown as well. ASPR, Age-Standardized Prevalence Rate; SDI, Sociodemographic Index; CI, concentration index.

As indicated by the slope index of inequality (SII), the disparities in ASPR, ASIR, and ASDR for headache disorders among countries have slightly increased over time, rising from 7,648.13 (95%CI: 6116.14, 9180.12), 2,506.76 (95%CI: 2046.45, 2967.06), and 88.45 (95%CI: 54.51, 122.38) in 1990 to 7,851.55 (95%CI: 6353.75, 9349.36), 2,557.94 (95%CI: 2091.11, 3024.78), and 100.38 (95%CI: 67.68, 133.07) in 2021 (Figure 7A; Supplementary Figures S11A, S12A). This suggests that countries with higher socio-economic status face a greater burden of headache disorders, with a marginal increase in burden disparities over the years. In contrast, the concentration index (CI) for ASPR, ASIR, and ASDR in 2021 were 0.05 (95%CI: 0.03–0.05), 0.05 (95%CI: 0.04–0.06), and 0.04 (95%CI: 0.03–0.05), respectively, showing no significant change compared to the values of 0.05 (95%CI: 0.04–0.05), 0.05 (95%CI: 0.04–0.06), and 0.04 (95%CI: 0.02–0.05) in 1990(Figure 7B; Supplementary Figures S11B, S12B). These values indicate that the burden of headache disorders remains skewed toward economically advantaged populations, while inequality in disease burden has remained largely unchanged over the three decades.

## Discussion

In 2015, the United Nations established Sustainable Development Goal 3 (SDG 3), with the objective to “Ensure healthy lives and promote well-being for all at all ages” (14). This goal sets forth a global commitment to decreasing mortality rates and disease burdens by 2030, highlighting the need for equitable healthcare access, preventive measures, and efficient disease management to enhance overall public health (15). Headache disorders are closely aligned with this goal due to their profound impact on individuals’ lives and their potential to cause complications, resulting in a substantial disease burden. Understanding the epidemiological trends of headache burdens is therefore essential to achieving health objectives. At present, some studies focus specifically on migraine or tension-type headache (7, 16, 17), while others target certain age groups or specific regions (18–20). However, these studies do not provide a comprehensive picture of the global burden and epidemiological trends of headache disorders as a whole. Comprehensive analyses that encompass prevalence, incidence, and DALYs across all countries and regions are still lacking. This gap limits the ability to fully grasp the global and regional dynamics of headache burden, making it challenging to tailor interventions effectively. Given these gaps, there is an urgent need to update the global data analysis of headache disorders. Such efforts can help policymakers better understand the healthcare environment and develop targeted prevention and intervention strategies to alleviate the burden of headache disorders and support the achievement of SDG 3.

Over the past 32 years, the global prevalence, incidence, and DALYs of headache disorders have increased significantly, with percentage changes of 57.16, 51.6, and 58.54%, respectively. Additionally, the global ASPR and ASDR have risen, while ASIR has declined. This could be linked to enhanced health awareness accompanying economic development (21). Additionally, this trend may be attributed to substantial progress in diagnosis, prevention, and prognosis, fueled by the clinical expertise gained by neurological healthcare professionals over the past 30 years. The advancement of advanced diagnostic and therapeutic technologies has also been instrumental in enhancing the management and understanding of headache disorders.

In 2021, the highest ASPR, ASIR, and ASDR were observed in high SDI regions, aligning with previous studies that have shown a greater burden of headache disorders in areas with higher SDI levels. However, the observed trends in the prevalence, incidence, and DALYs of headache disorders in this study deviate from the conventional assumptions about the relationship between disease burden and SDI. This suggests that the burden of headache disorders may be influenced by factors beyond those typically associated with SDI levels. Generally, regions with higher SDI levels tend to have better healthcare systems, leading to a decline in disease burden. However, in this study, the disease burden of headache disorders in high SDI regions remained the highest, consistent with findings from previous studies focusing on individuals aged 15–39 years (22). There may be two main reasons for the high prevalence of headache disorders in high SDI regions. On one hand, economic development has led to improved healthcare systems, resulting in higher diagnosis rates (7). On the other hand, changes in lifestyle may also contribute to the increase. Studies have shown that excessive use of electronic devices is associated with a higher risk of developing headache disorders (23). In addition, sleep, stress, emotions, and urban living all influence the occurrence of headaches (24–26). Additionally, local cultural norms and the stigma surrounding headache disorders further complicate efforts to manage the disease burden. These elements undermine the potential of sustained economic growth and social development to alleviate the impact of these conditions (27, 28). To reduce the high burden of headache disorders in high SDI regions, public health interventions should focus on raising awareness, improving diagnosis and treatment in primary care, and promoting healthy lifestyles such as better sleep and stress management. Challenges include overcoming cultural stigma, ensuring equitable access, and securing long-term funding and support.

Over the past 32 years, East Asia has seen the most rapid increases in ASPR, ASIR, and ASDR, likely influenced by factors including industrialization, urbanization, healthcare inequities, and air pollution (29, 30). In this study, the burden of headache disorders in Eastern Sub-Saharan Africa is shown to have decreased. However, a previous study indicates that the burden of migraine in the 15–39 age group in this region has increased (31). This discrepancy may be due to various factors, including improvements in diagnostic capabilities and healthcare access, which could lead to better identification and reporting of migraines in younger populations. Additionally, lifestyle changes, such as increased urbanization, stress, and changes in diet, could contribute to a rise in migraine burden in this specific age group, while other headache types may not show the same trend. Our study reveals considerable differences in the prevalence, incidence, and DALYs of headache disorders across various countries, indicating that the factors driving the burden of these disorders are complex and context-specific. Notably, Qatar demonstrated the largest percentage increases in the prevalence, incidence, and DALYs of headache disorders among the 204 countries studied, consistent with the global trend of rising disease burden (7). In contrast, the opposite trend seen in a few countries suggests that healthcare and public health initiatives have successfully mitigated headache disorder triggers and improved disease outcomes. Tracking these trends could offer valuable insights for shaping global strategies for headache disorder prevention and management. Furthermore, it is essential to implement targeted prevention and treatment strategies that address the specific burden of headache disorder in various populations.

In 2021, the burden of headache disorders exhibited a distinct age pattern, rising from adolescence and peaking in young adulthood. Adolescents experience unique pressures, such as heightened academic demands and rapid physical and psychological changes, especially during puberty. This emphasizes the need for focused attention and tailored health management strategies to meet the specific needs of this age group. The disease burden is consistently greater in females than in males, with a pronounced disparity among young women. This difference is driven by various factors, including socio-cultural influences, physical and emotional development, and hormonal fluctuations. Changes in sex hormones, particularly estrogen and progesterone, are key contributors to the onset of headache disorder in women (32). Young women of childbearing age, particularly those balancing emotional, romantic, and reproductive responsibilities, are more vulnerable to emotional fluctuations that can trigger headache disorders. Furthermore, women in this demographic frequently manage multiple roles, such as work, caregiving, and childcare, requiring significant energy and emotional resilience (33). These responsibilities can lead to increased fatigue and anxiety, which in turn heighten the frequency of headaches and raise the risk of complications. Women tend to have lower thresholds for stress and pain compared to men, making them more susceptible to headache disorder. The elevated prevalence and frequency of headache disorder in women have reinforced the perception of this condition as gender-specific. For example, certain pharmaceutical marketing campaigns categorize headache disorder as a “women’s disease,” which may perpetuate gender bias and hinder female patients from receiving adequate care and support (34). In addition, the overall burden of headache disorders in the elderly is lower than that in younger people. On one hand, this may be because elderly individuals face less social pressure compared to younger people. On the other hand, the diagnosis of headache disorders in the elderly population faces more challenges. To address gender and age differences in headache disorders, public health strategies should focus on three areas: prioritize resources for adolescents and women of childbearing age through specialized clinics and stronger primary care; raise awareness about hormone-related triggers and promote stress management via schools and communities; and improve diagnosis by training providers on gender- and age-specific symptoms and creating simple screening tools for the elderly. These steps can reduce headache burden and health inequalities effectively.

Our decomposition analysis identified an interesting trend: between 1990 and 2021, the overall burden of headache disorders declined in both Central and Eastern Europe. This decline suggests potential progress in healthcare management, prevention strategies, and lifestyle adjustments in these regions during this period. Several factors may contribute to this trend, including declining birth rates and an aging population (35). Previous findings have indicated that young people bear a greater burden of headaches; thus, a reduction in the proportion of young people within the overall population could directly lead to a decrease in headache burden. Furthermore, several of these regions have undergone rapid economic development and substantial improvements in healthcare infrastructure, leading to enhanced access to medical services, increased awareness, and better diagnostic capabilities (36). These advancements have likely played a role in more effective headache disorder management, thereby reducing the overall burden. However, despite the observed decline, these regions still exhibit relatively high headache disorder burdens compared to global averages. Continued research is crucial to understand the factors driving these regional reductions and to assess whether the strategies employed in these regions can be adapted to help alleviate the global headache disorder burden, particularly among younger populations.

Inequality analysis reveals that the burden of headache disorders is consistently disproportionate with socio-economic development. The disease burden tends to concentrate in economically developed countries, a trend that has remained largely unchanged over the past 32 years. This may be attributed to the demands of economic growth, requiring individuals to devote more energy to work, increasing competitive pressure, and reducing rest time. Simultaneously, healthcare systems may not have adequately improved to address the rising burden of disease. These findings provide valuable guidance for policymakers to prioritize targeted interventions and healthcare system enhancements to address this growing inequality effectively.

This study highlights the growing global burden of headache disorders, emphasizing their profound impact on health systems and individual well-being. The findings underscore the importance of targeted public health strategies to address the increasing prevalence, incidence, and DALYs, particularly in high-SDI regions and among women of reproductive age. Policymakers can leverage these insights to design interventions that prioritize high-burden populations and regions, while addressing disparities. Additionally, the observed trends call for investments in healthcare systems, improving diagnostic capabilities, and developing preventive measures to alleviate the disease burden.

## Limitations

This study has several limitations. First, the data were sourced from the GBD 2021, which relies on various data sources and modeling methods, potentially introducing biases or inaccuracies in regions with limited data availability. Second, while the study provides comprehensive global estimates, it does not account for potential underdiagnosis or cultural differences in headache disorder reporting, particularly in low-SDI regions. Third, Due to limitations in the GBD dataset, the lack of external validation, sensitivity analyses, and adjustment for key confounders such as healthcare access, diagnostic practices, and cultural factors may affect the interpretation of our findings. Future research should integrate primary data collection to address these gaps and validate findings.

## Conclusion

Between 1990 and 2021, the global burden of headache disorders rose significantly, highlighting notable disparities across SDI regions, countries, age groups, and genders. High SDI regions reported the highest ASPR, ASIR, and ASDR, while Middle SDI regions saw the most rapid increases. Globally, adolescents and young adults experienced the greatest burden, with females consistently facing a higher burden than males. Contributing factors to the growing headache disorder burden in certain areas include rapid urbanization, economic growth, and high-stress lifestyles. Economically advanced regions exhibited a disproportionately higher burden of headache disorders.

## Data Availability

The original contributions presented in the study are included in the article/Supplementary material, further inquiries can be directed to the corresponding author.

## Footnotes

^1^ https://ghdx.healthdata.org/gbd-2021/sources
