# Supplementary material for: Global trends and regional disparities in the burden of headache disorders, 1990–2021: a comprehensive analysis of the global burden of disease study
Source: Front Neurol. 2025 Jun 5;16:1575705. doi: 10.3389/fneur.2025.1575705 (PMC12176582; doi:10.3389/fneur.2025.1575705)
Supplement: Supplementary file 1 [file Table_1.docx]

**Table S1.** Incidence of headache disorder between 1990-2021 at the global and regional level

| Location | 1990 |  | 2021 |  | 1990-  2021 |  |
| --- | --- | --- | --- | --- | --- | --- |
|  | **Number**  **(95%UI)** | **ASR**  **(95%UI)** | **Number**  **(95%UI)** | **ASR**  **(95%UI)** | **Cases change**  **(95%UI)** | **EAPC_95%CI** |
| Global | 533794823.90(472464996.93,591430931.57) | 10097.25(8965.19,11186.25) | 809226480.19(717818771.04,895990201.46) | 10084.51(8956.48,11170.76) | 51.60(48.08,54.84) | -0.0002(-0.01,0.01) |
| High SDI | 110194635.29(97431274.86,122726723.51) | 12124.37(10692.14,13476.79) | 133602787.02(118033322.13,147751977.21) | 11998.50(10585.06,13303.17) | 21.24(18.12,24.53) | -0.05(-0.06,-0.04) |
| High-middle SDI | 106341016.26(93968679.21,118115375.83) | 9755.30(8652.50,10822.83) | 130725735.58(115265920.91,144453096.02) | 9713.78(8613.29,10776.68) | 22.93(18.40,27.37) | 0.01(-0.01,0.04) |
| Middle SDI | 158808170.63(140108526.68,176639687.52) | 9217.49(8180.97,10218.00) | 243446537.63(215909105.51,269427846.80) | 9683.67(8598.09,10721.40) | 53.30(47.22,58.94) | 0.17(0.16,0.19) |
| Low-middle SDI | 114900538.10(101198502.60,127510606.19) | 10316.42(9153.81,11395.80) | 200895653.98(177803496.56,222958166.99) | 10293.60(9125.41,11392.13) | 74.84(71.05,78.80) | -0.02(-0.03,-0.01) |
| Low SDI | 43014225.18(37852915.70,48110769.49) | 9454.00(8371.23,10501.68) | 99897406.67(87792677.93,111753942.33) | 9366.36(8294.27,10401.70) | 132.24(130.28,134.16) | -0.04(-0.05,-0.03) |
| Andean Latin America | 3171010.77(2789098.23,3558984.72) | 8439.41(7488.92,9324.07) | 5659403.61(4986233.98,6289281.70) | 8441.81(7468.56,9345.49) | 78.47(70.76,86.07) | 0.01(0.00,0.02) |
| Australasia | 2281530.82(2020125.19,2537950.76) | 10898.14(9622.51,12079.50) | 3436041.34(3026090.03,3801502.06) | 10895.04(9610.14,12058.95) | 50.60(46.91,53.98) | 0.00(-0.00,0.00) |
| Caribbean | 3425865.56(3008494.20,3817880.17) | 9707.17(8580.63,10802.80) | 4671094.00(4127092.68,5182029.42) | 9700.89(8578.68,10811.34) | 36.35(32.11,40.59) | -0.00(-0.00,-0.00) |
| Central Asia | 7931183.97(6892506.74,8895988.24) | 11774.08(10272.74,13164.67) | 11296260.13(9822321.11,12718807.60) | 11764.53(10241.37,13153.90) | 42.43(38.32,46.68) | -0.00(-0.00,-0.00) |
| Central Europe | 15315754.89(13431788.23,17025334.89) | 11927.39(10473.43,13281.88) | 14086702.42(12455869.12,15580372.60) | 11943.96(10495.46,13312.91) | -8.02(-10.81,-5.34) | 0.00(0.00,0.00) |
| Central Latin America | 16134692.76(14187777.10,18039966.03) | 9984.35(8842.42,11070.16) | 25974342.07(23026886.05,28826696.83) | 9989.58(8859.54,11075.49) | 60.98(54.85,67.08) | 0.00(-0.00,0.00) |
| Central Sub-Saharan Africa | 4630529.85(4026286.94,5223521.70) | 9363.16(8230.75,10403.03) | 12109701.44(10544193.70,13654756.26) | 9360.43(8235.58,10399.97) | 161.52(159.75,163.19) | -0.00(-0.00,-0.00) |
| East Asia | 91601537.84(80589264.10,102626678.36) | 7391.25(6542.16,8251.16) | 119035994.01(105291261.52,132586261.02) | 7816.63(6900.54,8698.47) | 29.95(22.16,37.14) | 0.22(0.17,0.26) |
| Eastern Europe | 28300742.18(25074997.38,31500582.63) | 12232.45(10807.82,13582.62) | 25940760.78(22902462.71,28699839.74) | 12263.83(10834.87,13644.35) | -8.34(-11.30,-5.13) | 0.01(-0.00,0.01) |
| Eastern Sub-Saharan Africa | 13434870.96(11737848.96,15225201.03) | 7902.36(6962.17,8827.45) | 31589589.58(27578741.66,35627163.98) | 7797.13(6869.75,8703.11) | 135.13(132.74,137.69) | -0.06(-0.08,-0.05) |
| High-income Asia Pacific | 20985917.10(18605813.70,23293180.11) | 11523.21(10206.32,12728.80) | 21669335.37(19154376.68,23944763.22) | 11465.19(10094.65,12740.55) | 3.26(-1.09,7.88) | -0.02(-0.02,-0.01) |
| High-income North America | 38913911.23(34245826.74,43488124.84) | 13478.43(11862.57,15038.09) | 50025407.65(44488083.68,55381393.83) | 13330.95(11802.72,14776.65) | 28.55(24.17,33.82) | -0.04(-0.05,-0.03) |
| North Africa and Middle East | 31827864.25(27914565.63,35708275.79) | 9843.83(8746.11,10886.60) | 62121674.87(55272620.79,69280987.48) | 9873.96(8763.15,10927.05) | 95.18(87.83,102.31) | 0.02(0.00,0.03) |
| Oceania | 571085.60(502430.07,641108.54) | 9160.19(8099.59,10168.20) | 1234791.19(1090287.07,1386828.75) | 9163.96(8100.85,10179.29) | 116.22(112.93,119.87) | 0.00(0.00,0.00) |
| South Asia | 111596403.13(98547825.93,124222325.59) | 10553.53(9368.44,11658.64) | 201652156.51(178711512.31,224063361.00) | 10562.40(9357.97,11658.05) | 80.70(76.47,85.02) | -0.01(-0.03,0.01) |
| Southeast Asia | 47660971.53(41875697.41,53178429.58) | 10364.14(9186.80,11498.43) | 74413839.85(65875116.25,82939949.11) | 10366.71(9186.59,11502.94) | 56.13(50.70,61.51) | 0.00(-0.00,0.00) |
| Southern Latin America | 5143892.46(4543764.86,5738089.01) | 10397.52(9222.29,11603.22) | 7317406.30(6461395.83,8147674.98) | 10441.24(9236.06,11649.38) | 42.25(39.04,45.26) | 0.02(0.02,0.02) |
| Southern Sub-Saharan Africa | 4951264.62(4351689.82,5550063.13) | 9824.10(8703.05,10964.42) | 8036087.69(7102963.48,9035493.51) | 9815.10(8699.55,10952.66) | 62.30(57.46,67.30) | -0.00(-0.00,-0.00) |
| Tropical Latin America | 18093146.92(16081742.62,20057580.68) | 11588.15(10425.49,12758.27) | 27140305.15(24259183.84,30053622.78) | 11823.80(10533.68,13019.34) | 50.00(44.03,56.52) | 0.05(0.04,0.07) |
| Western Europe | 50122965.90(44386823.66,55958684.06) | 12650.14(11113.06,14117.58) | 55477379.37(49552163.08,61469217.94) | 12661.01(11128.29,14103.83) | 10.68(7.96,13.20) | 0.02(0.01,0.02) |
| Western Sub-Saharan Africa | 17699681.55(15607936.70,19738259.07) | 10187.76(9018.38,11328.27) | 46338206.85(40696774.36,51748717.48) | 10184.19(9007.95,11310.80) | 161.80(160.38,163.15) | -0.01(-0.02,0.00) |

**Abbreviations:** EAPC, estimated annual percentage change; SDI, sociodemographic Index; UI, uncertainty interval; CI, confidence interval; ASR, age-standardized rates.

**Table S2.** DALYs of headache disorder between 1990-2021 at the global and regional level

| Location | 1990 |  | 2021 |  | 1990-  2021 |  |
| --- | --- | --- | --- | --- | --- | --- |
|  | **Number**  **(95%UI)** | **ASR**  **(95%UI)** | **Number**  **(95%UI)** | **ASR**  **(95%UI)** | **Cases change**  **(95%UI)** | **EAPC_95%CI** |
| Global | 30260883.92(5963391.97,64833434.46) | 583.75(122.49,1231.64) | 47975675.06(9800212.26,100667852.52) | 588.39(117.58,1245.36) | 58.54(54.24,65.71) | 0.04(0.03,0.05) |
| High SDI | 6026677.38(1355771.39,12503912.46) | 641.20(138.26,1342.85) | 7453720.63(1787749.84,15361008.41) | 640.40(135.42,1337.70) | 23.68(19.71,32.92) | 0.01(-0.02,0.04) |
| High-middle SDI | 6346250.22(1590842.32,13160367.03) | 574.78(146.54,1189.21) | 8318285.25(2150201.20,17015818.44) | 577.50(134.57,1201.76) | 31.07(25.08,37.94) | 0.06(0.04,0.07) |
| Middle SDI | 9398627.24(1617027.09,20359640.16) | 557.77(104.98,1185.18) | 15284069.03(2903732.51,32354748.10) | 526.97(112.22,1115.94) | 62.62(55.83,82.21) | 0.18(0.17,0.20) |
| Low-middle SDI | 6268038.80(1006092.11,13759643.44) | 596.36(107.32,1277.90) | 11616300.11(1981001.52,25231490.85) | 597.24(107.79,1280.45) | 85.33(79.69,100.67) | 0.00(-0.01,0.01) |
| Low SDI | 2191131.09(407075.64,4757189.85) | 524.09(112.16,1103.07) | 5264012.68(973047.30,11537810.89) | 526.97(112.22,1115.94) | 140.24(135.26,144.64) | 0.02(0.02,0.03) |
| Andean Latin America | 151608.62(32428.65,325543.40) | 427.77(105.31,887.49) | 304989.10(70250.46,645666.93) | 449.99(105.16,944.58) | 101.17(89.46,120.30) | 0.19(0.15,0.24) |
| Australasia | 119886.57(28232.47,246198.75) | 557.57(127.88,1154.40) | 183163.26(45149.19,374049.13) | 559.63(127.13,1152.04) | 52.78(47.34,64.17) | 0.01(0.01,0.01) |
| Caribbean | 203829.70(34141.05,437111.54) | 583.51(106.44,1249.12) | 284205.47(53793.36,603515.84) | 580.20(105.83,1236.46) | 39.43(34.32,58.90) | -0.01(-0.02,-0.01) |
| Central Asia | 369689.87(79489.25,796991.46) | 576.87(135.47,1220.10) | 559509.46(131253.45,1181478.15) | 574.24(134.70,1212.09) | 51.35(46.27,64.15) | -0.01(-0.01,-0.00) |
| Central Europe | 796588.66(224082.93,1663178.84) | 599.99(162.56,1264.45) | 768193.81(238261.14,1575941.56) | 599.93(163.52,1264.19) | -3.56(-7.28,5.31) | 0.01(0.00,0.01) |
| Central Latin America | 893845.03(147620.94,1925654.30) | 578.37(112.71,1229.31) | 1548270.89(300349.99,3319150.68) | 585.10(112.90,1255.46) | 73.21(64.88,103.68) | 0.05(0.04,0.06) |
| Central Sub-Saharan Africa | 231826.17(45546.68,499754.51) | 511.90(114.90,1058.10) | 617713.07(121077.26,1311959.21) | 515.36(117.70,1065.71) | 166.46(159.48,174.55) | 0.03(0.03,0.04) |
| East Asia | 5729155.21(1124057.33,12243945.69) | 456.25(93.59,963.99) | 7988579.82(1685809.13,16763663.04) | 487.69(95.05,1037.09) | 39.44(29.23,55.20) | 0.25(0.21,0.29) |
| Eastern Europe | 1638231.30(614163.11,3222353.20) | 671.97(243.18,1336.61) | 1576590.49(612730.26,3028642.64) | 671.26(241.79,1331.61) | -3.76(-6.53,2.57) | 0.05(0.00,0.09) |
| Eastern Sub-Saharan Africa | 592409.59(149580.95,1283854.28) | 391.59(112.52,811.36) | 1459522.92(362961.38,3168300.35) | 395.30(112.26,821.89) | 146.37(139.38,150.92) | 0.06(0.04,0.07) |
| High-income Asia Pacific | 918151.88(256614.97,1913714.90) | 483.34(129.95,1016.50) | 967560.53(299105.09,1956005.21) | 486.20(128.87,1017.31) | 5.38(-0.93,18.77) | 0.01(0.00,0.03) |
| High-income North America | 2102176.07(425409.32,4429862.84) | 705.13(138.00,1496.34) | 2656814.16(581261.93,5580169.63) | 684.01(135.48,1445.40) | 26.38(20.80,38.44) | -0.04(-0.12,0.03) |
| North Africa and Middle East | 2039502.88(441143.26,4382999.88) | 665.47(162.62,1370.67) | 4267741.55(1031392.75,8814827.55) | 666.72(161.94,1371.46) | 109.25(100.63,130.53) | 0.01(-0.00,0.02) |
| Oceania | 33221.68(4725.52,72807.05) | 563.40(93.26,1200.92) | 74707.08(11345.36,161838.02) | 565.23(93.25,1210.06) | 124.87(118.85,136.93) | 0.01(0.01,0.01) |
| South Asia | 5855984.56(898725.89,13006928.23) | 585.11(100.86,1270.23) | 11316917.91(1831990.37,24611220.79) | 588.86(100.40,1268.61) | 93.25(85.77,108.32) | 0.00(-0.02,0.03) |
| Southeast Asia | 2946195.26(387795.43,6364813.74) | 667.84(99.37,1430.10) | 4881346.06(745495.37,10341105.81) | 658.16(99.35,1400.95) | 65.68(59.58,89.74) | -0.04(-0.05,-0.03) |
| Southern Latin America | 240917.30(60185.94,501287.31) | 492.64(125.99,1019.04) | 363162.86(94946.81,736395.10) | 502.39(127.00,1027.89) | 50.74(44.47,59.20) | 0.10(0.08,0.12) |
| Southern Sub-Saharan Africa | 254586.68(52312.38,538374.46) | 541.35(126.26,1106.82) | 434222.35(97467.90,893645.97) | 534.51(125.58,1092.86) | 70.56(65.49,84.69) | -0.04(-0.04,-0.03) |
| Tropical Latin America | 1113400.61(137154.28,2450337.66) | 720.52(103.53,1552.08) | 1732233.42(262708.67,3682241.80) | 735.36(103.39,1570.72) | 55.58(46.71,90.91) | 0.14(0.10,0.17) |
| Western Europe | 3049027.20(640051.60,6370410.48) | 744.31(145.59,1581.07) | 3387292.30(764627.48,6980774.17) | 748.58(144.83,1578.77) | 11.09(7.47,20.19) | 0.05(0.02,0.08) |
| Western Sub-Saharan Africa | 980649.09(169097.26,2163552.24) | 604.56(122.01,1290.70) | 2602938.56(444801.26,5761190.89) | 611.98(123.47,1311.20) | 165.43(158.99,170.85) | 0.05(0.04,0.05) |

**Abbreviations:** EAPC, estimated annual percentage change; SDI, sociodemographic Index; UI, uncertainty interval; CI, confidence interval; ASR, age-standardized rates.
