# Supplementary material for: Global trends and regional disparities in the burden of headache disorders, 1990–2021: a comprehensive analysis of the global burden of disease study
Source: Front Neurol. 2025 Jun 5;16:1575705. doi: 10.3389/fneur.2025.1575705 (PMC12176582; doi:10.3389/fneur.2025.1575705)
Supplement: Supplementary file 2 [file Table_2.docx]

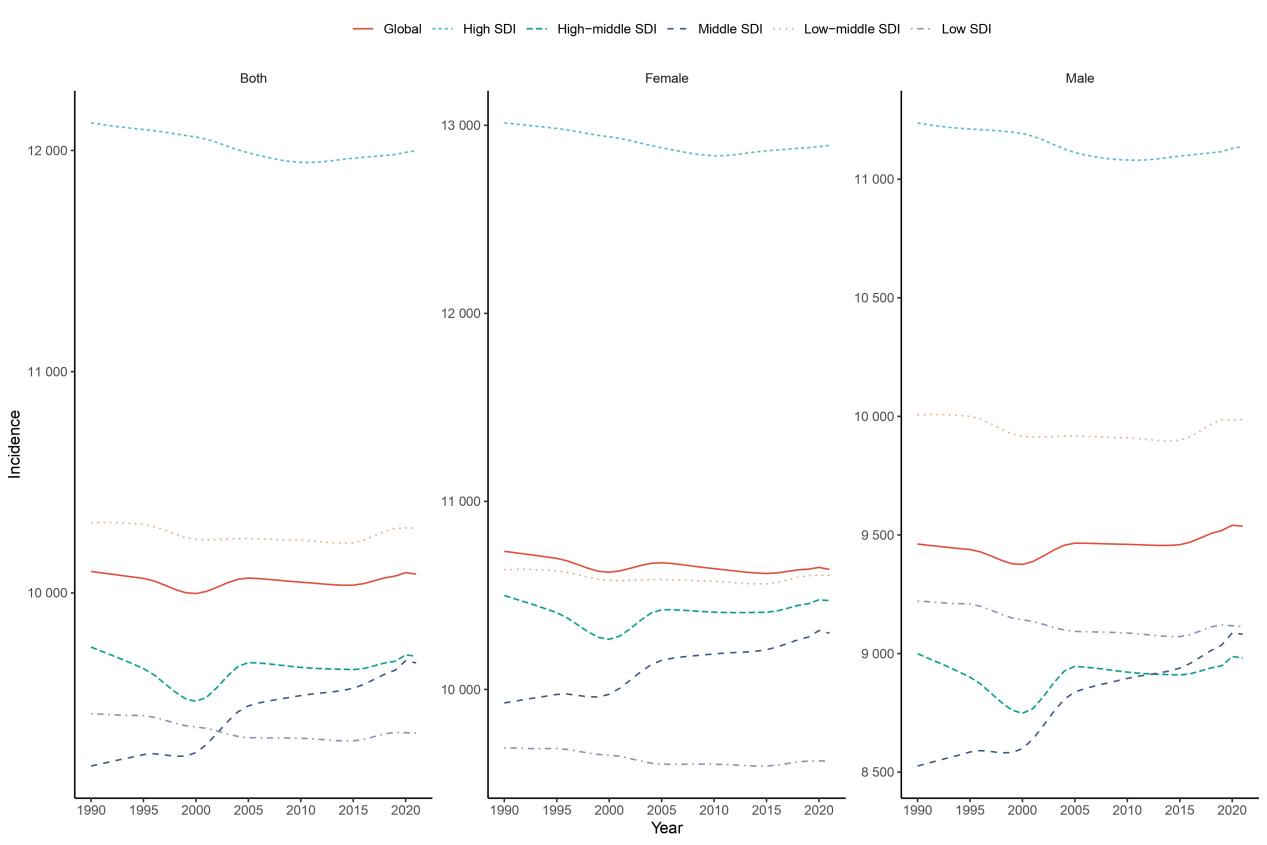


**Figure S1.** Trends of incidence in headache disorder from 1990 to 2021

**Figure legend:** Figure S1 illustrates the global trends in ASIR of headache disorders from 1990 to 2021, stratified by SDI regions and sex. Panel A shows data for both sexes combined, Panel B for females, and Panel C for males. High SDI regions consistently displayed the highest ASIR across the study period, while low SDI regions exhibited the lowest rates. Female ASIR was higher than male ASIR across all SDI regions.

**Abbreviations:** SDI, Socio-demographic Index; ASIR, Age-Standardized Incidence Rate.


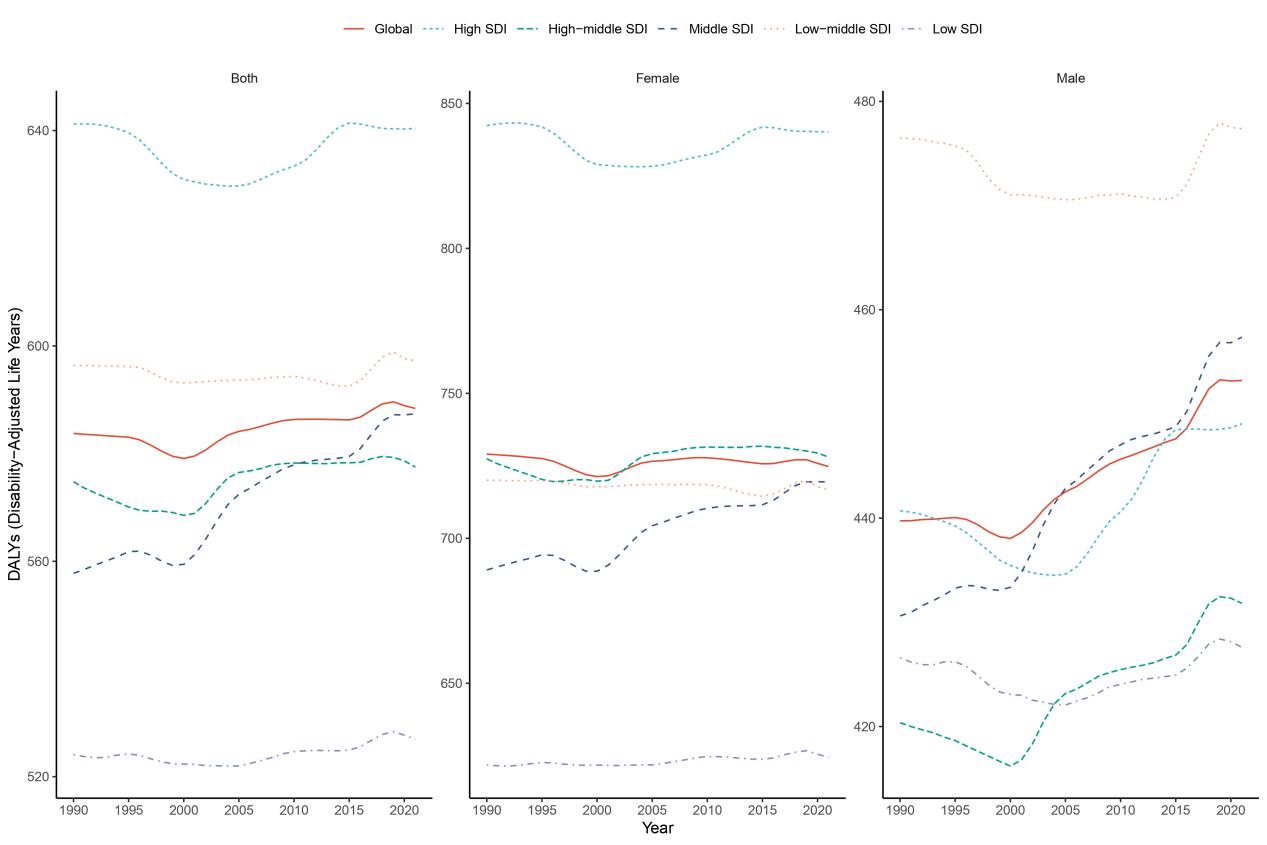


**Figure S2.** Trends of DALYs in headache disorder from 1990 to 2021

**Figure legend:** Figure S2 presents the trends in ASDR of headache disorders from 1990 to 2021, stratified by SDI regions and sex. Panel A shows data for both sexes combined, Panel B for females, and Panel C for males. High SDI regions consistently displayed the highest ASDR, while low SDI regions reported the lowest rates. Females experienced higher DALY rates than males across all SDI regions.

**Abbreviations:** SDI, Socio-demographic Index; DALY, Disability-Adjusted Life Year; ASDR, Age-Standardized DALY Rate.


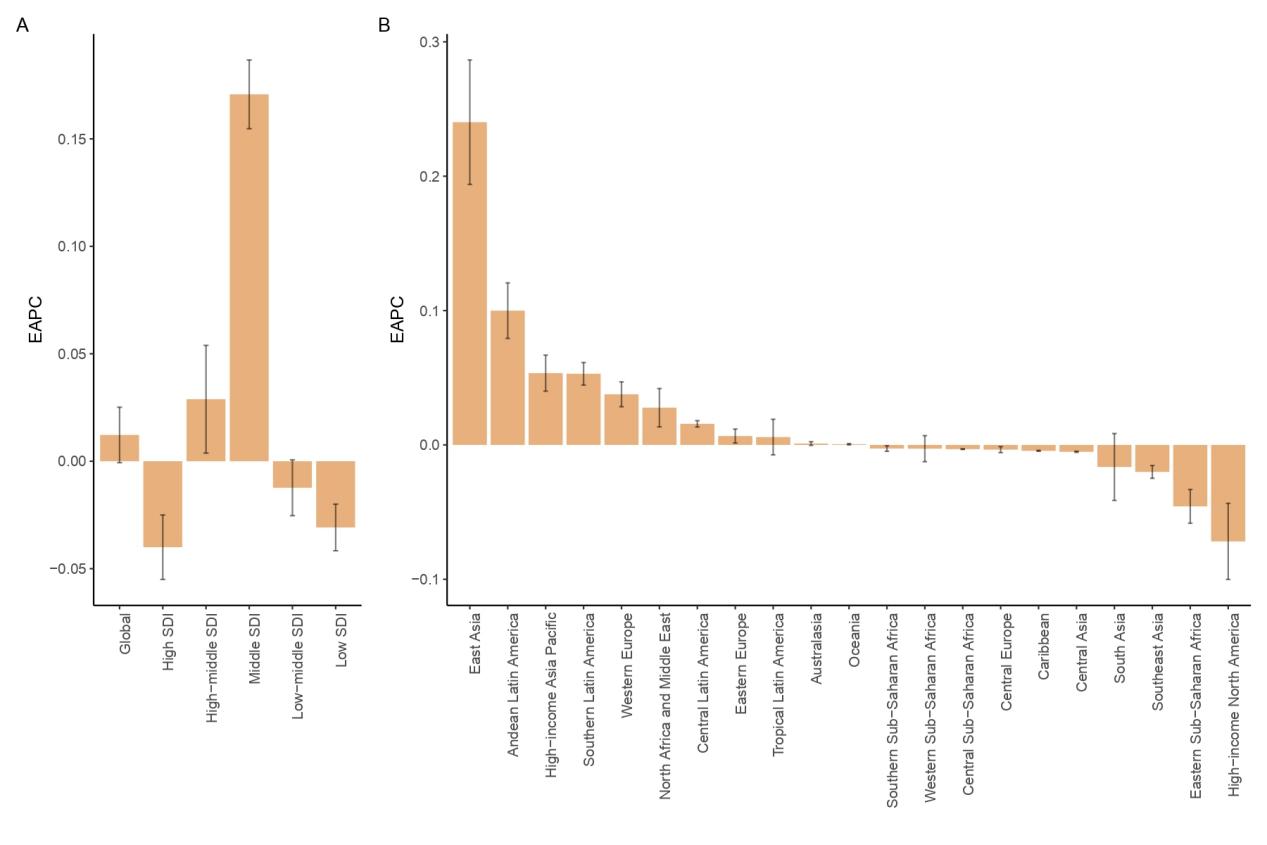


**Figure S3. A** EAPC for the ASPR of headache disorder across global and 5 SDI regions. **B** EAPC for the ASPR of headache disorder across 21 GBD regions.

**Figure legend:** (A) The EAPC for global and regional headache disorder ASPR from 1990 to 2021. The bars represent the EAPC with error bars indicating the 95% confidence intervals across regions and SDI categories. (B) The detailed EAPC values for various regions ranked by their SDI, highlighting the trends in global and regional ASPR. Regions with positive values show an increasing trend, whereas negative values indicate a decrease in ASPR.

**Abbreviations:** ASPR: Age-Standardized Prevalence Rate; EAPC: Estimated Annual Percentage Change; SDI: Socio-Demographic Index


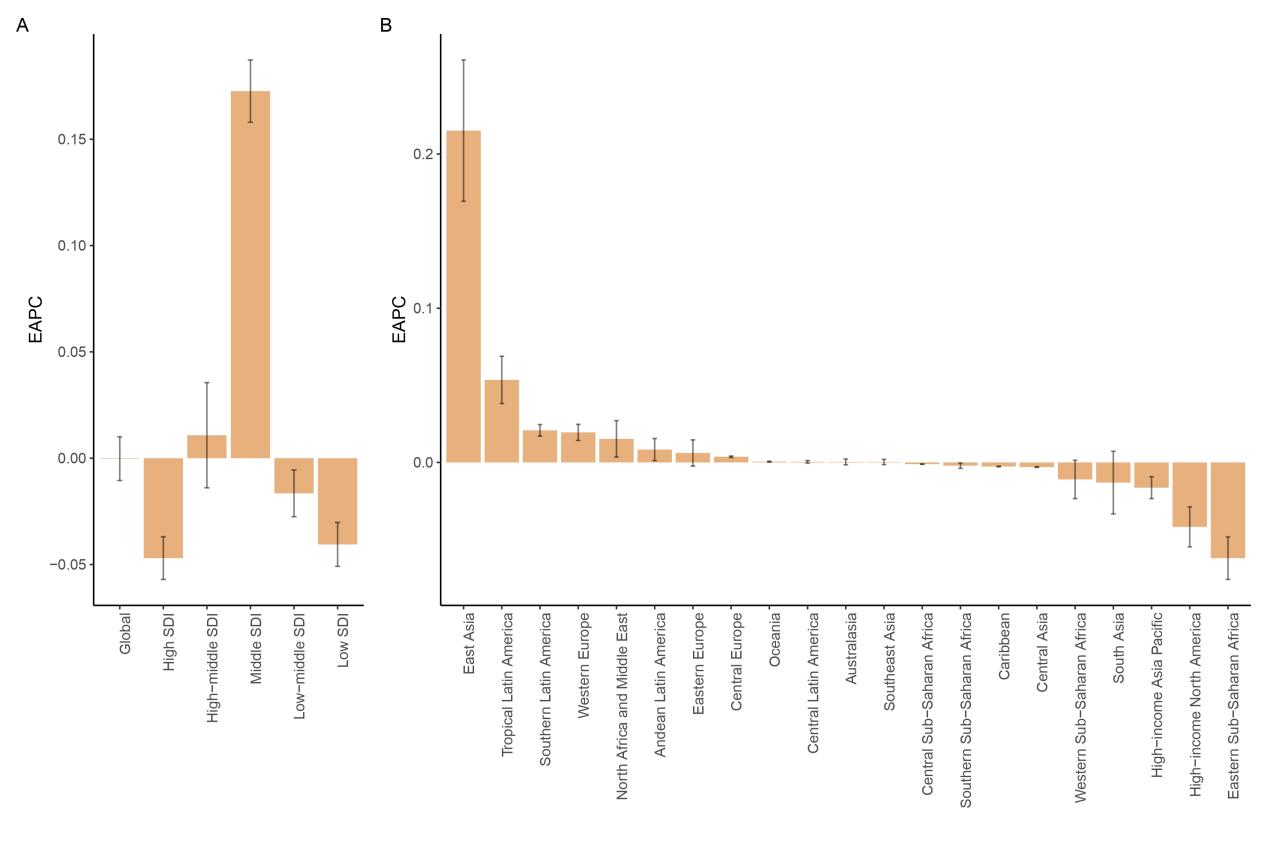


**Figure S4. A** EAPC for the ASIR of headache disorder across global and 5 SDI regions. **B** EAPC for the ASIR of headache disorder across 21 GBD regions.

**Figure legend:** (A) The EAPC for global and regional headache disorder ASIR from 1990 to 2021. The bars represent the EAPC with error bars indicating the 95% confidence intervals across regions and SDI categories. (B) The detailed EAPC values for various regions ranked by their SDI, highlighting the trends in global and regional ASIR. Regions with positive values show an increasing trend, whereas negative values indicate a decrease in ASIR.

**Abbreviations:** ASIR, Age-Standardized incidence Rate; EAPC, Estimated Annual Percentage Change; SDI, Socio-Demographic Index


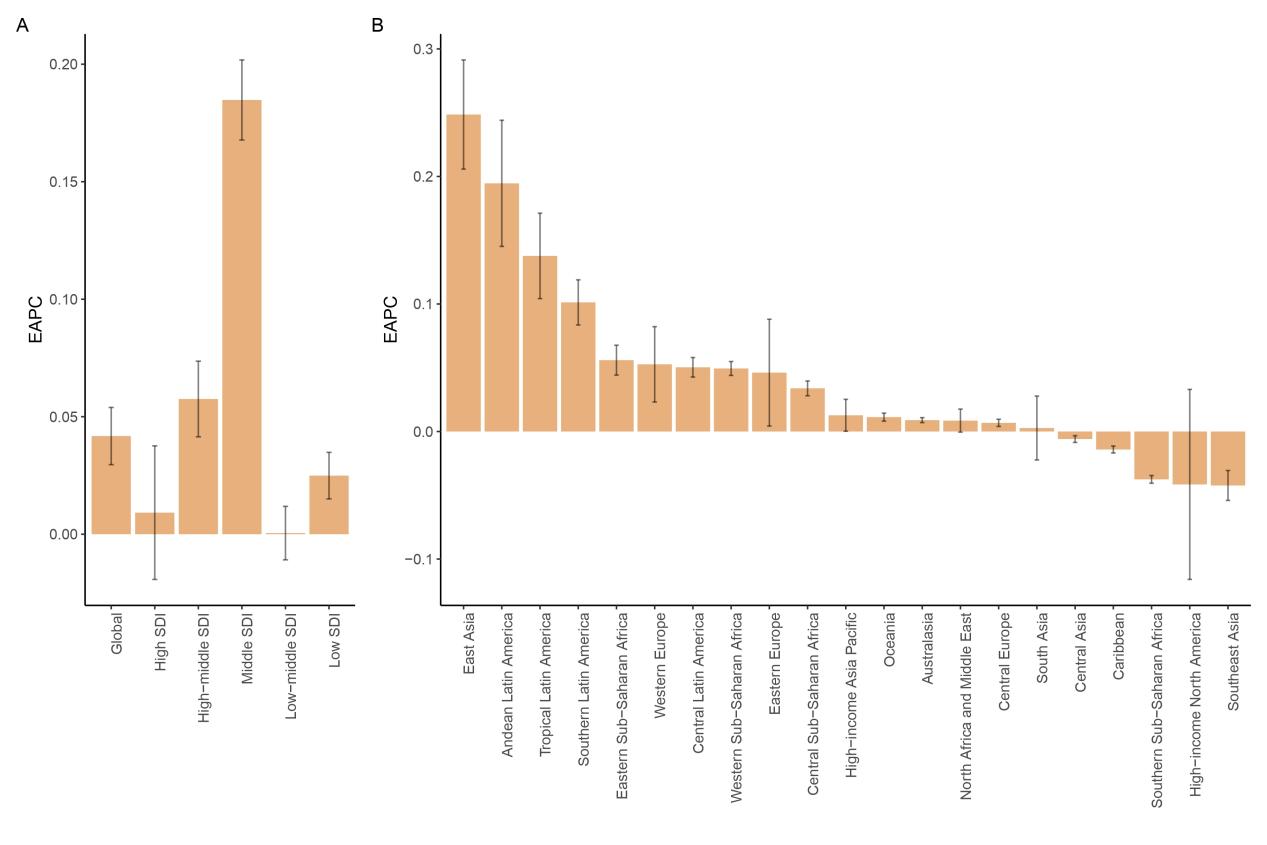


**Figure S5. A** EAPC for the ASDR of headache disorder across global and 5 SDI regions. **B** EAPC for the ASDR of headache disorder across 21 GBD regions.

**Figure legend:** (A) The EAPC for global and regional headache disorder ASDR from 1990 to 2021. The bars represent the EAPC with error bars indicating the 95% confidence intervals across regions and SDI categories. (B) The detailed EAPC values for various regions ranked by their SDI, highlighting the trends in global and regional ASDR. Regions with positive values show an increasing trend, whereas negative values indicate a decrease in ASDR.

**Abbreviations:** ASDR, Age-Standardized DALYs Rate; EAPC, Estimated Annual Percentage Change; SDI, Socio-Demographic Index


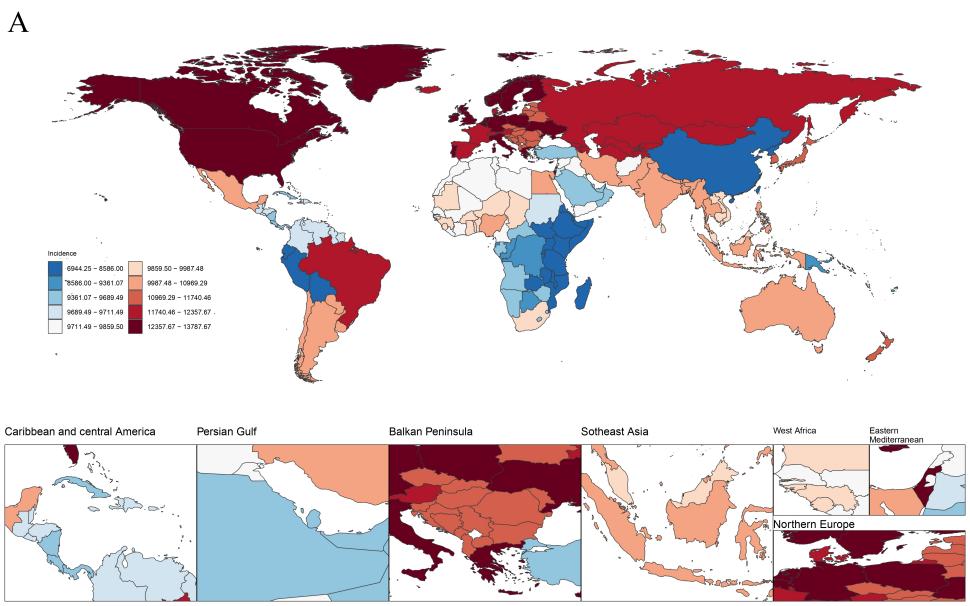


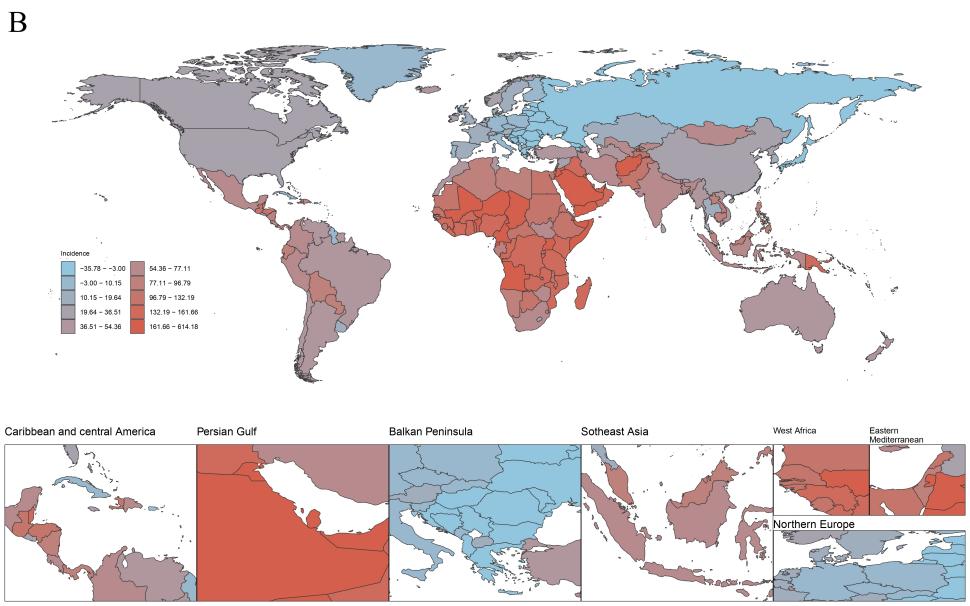


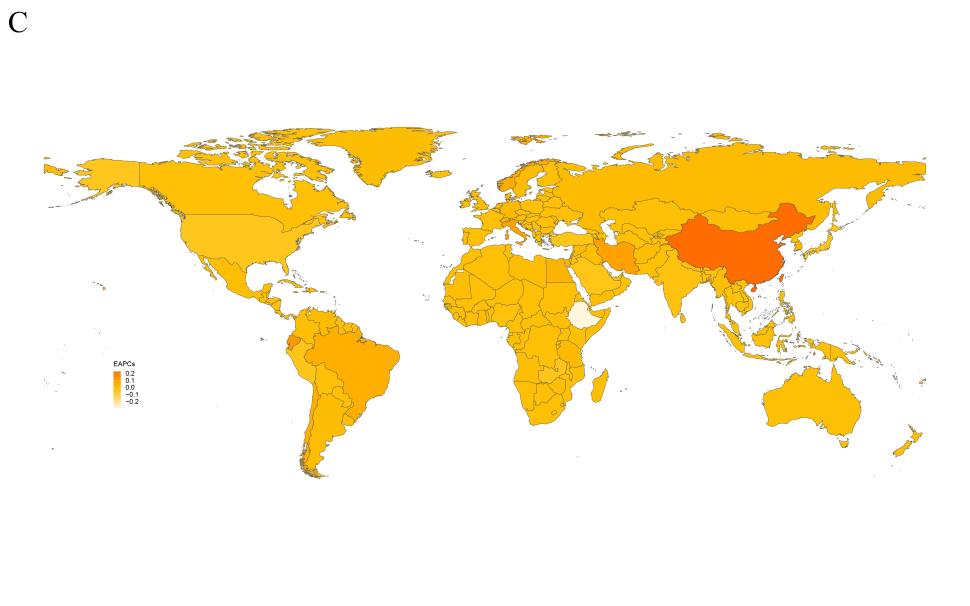


**Figure S6. A** The ASIR of headache disorders in 204 countries and territories in 2021. **B** The case changes in the incidence of headache disorders across 204 countries and territories from 1990 to 2021. **C** EAPC of ASIR.

**Figure legend:**

1. Global ASIR of headache disorders per 100,000 population in 2021. Darker shades indicate higher incidence, with detailed regional focus in Caribbean and Central America, Persian Gulf, Balkan Peninsula, Southeast Asia, West Africa, Eastern Mediterranean, and Northern Europe.
2. Percent change in incidence rates of headache disorders from 1990 to 2021, showing areas with significant increases or decreases.
3. EAPCs of ASIR of headache disorder from 1990 to 2021, highlighting regions with notable growth or decline.

**Abbreviations:** ASIR, age-standardized incidence rate; EAPC, estimated annual percentage change.


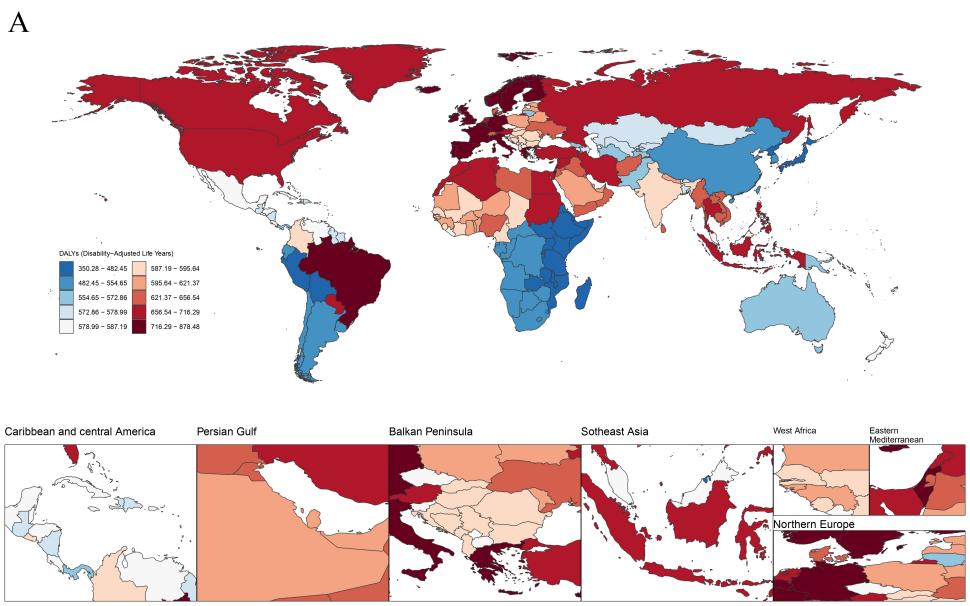


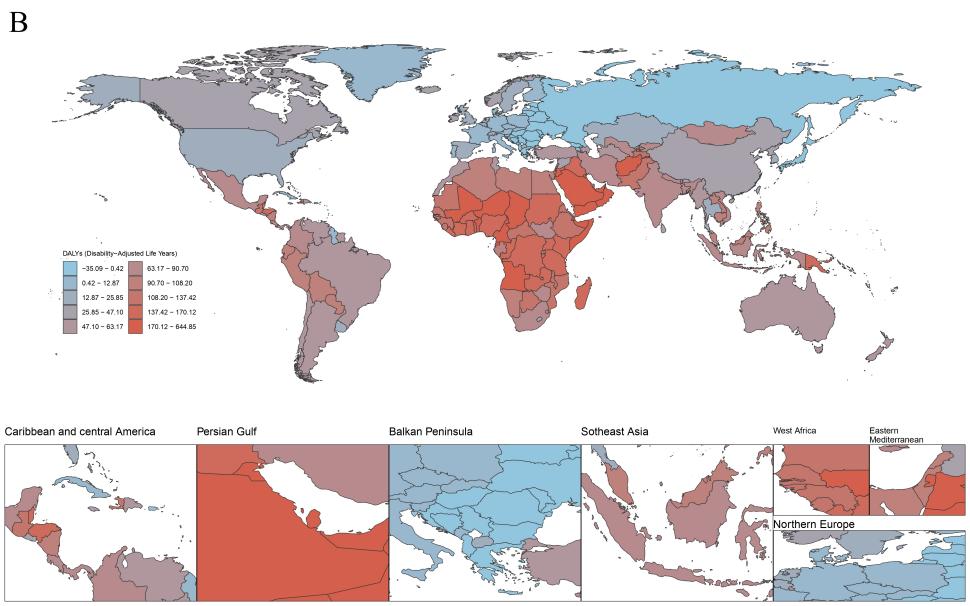


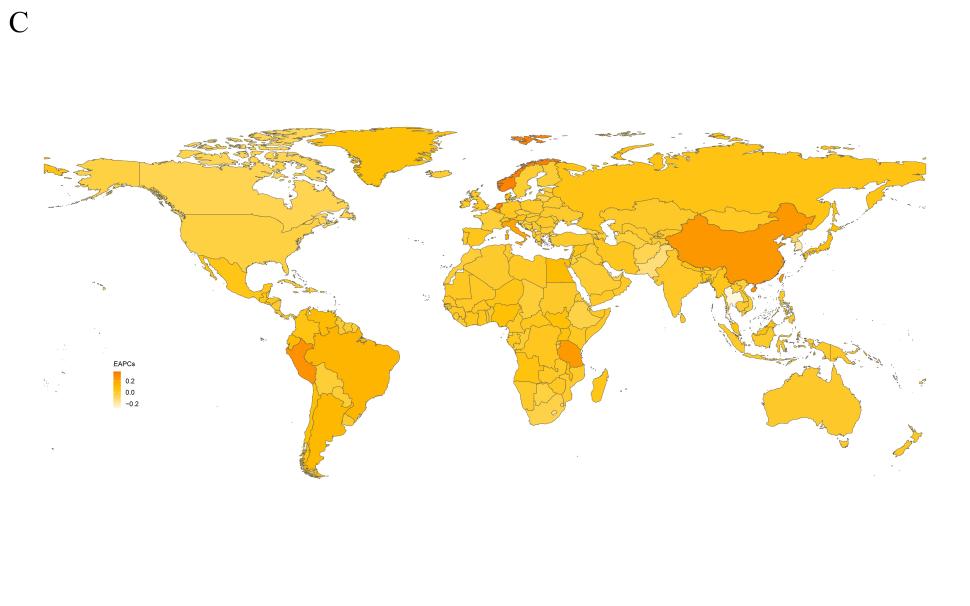


**Figure S7.** **A** The ASDR of headache disorders in 204 countries and territories in 2021. **B** The case changes in the DALYs of headache disorders across 204 countries and territories from 1990 to 2021. **C** EAPC of ASDR.

**Figure legend:**

1. Global ASDR of headache disorders per 100,000 population in 2021. Darker shades indicate higher DALYs, with detailed regional focus in Caribbean and Central America, Persian Gulf, Balkan Peninsula, Southeast Asia, West Africa, Eastern Mediterranean, and Northern Europe.
2. Percent change in DALYs rates of headache disorders from 1990 to 2021, showing areas with significant increases or decreases.
3. EAPCs of ASDR of headache disorder from 1990 to 2021, highlighting regions with notable growth or decline.

**Abbreviations:** ASDR, age-standardized DALYs rate; EAPC, estimated annual percentage change.


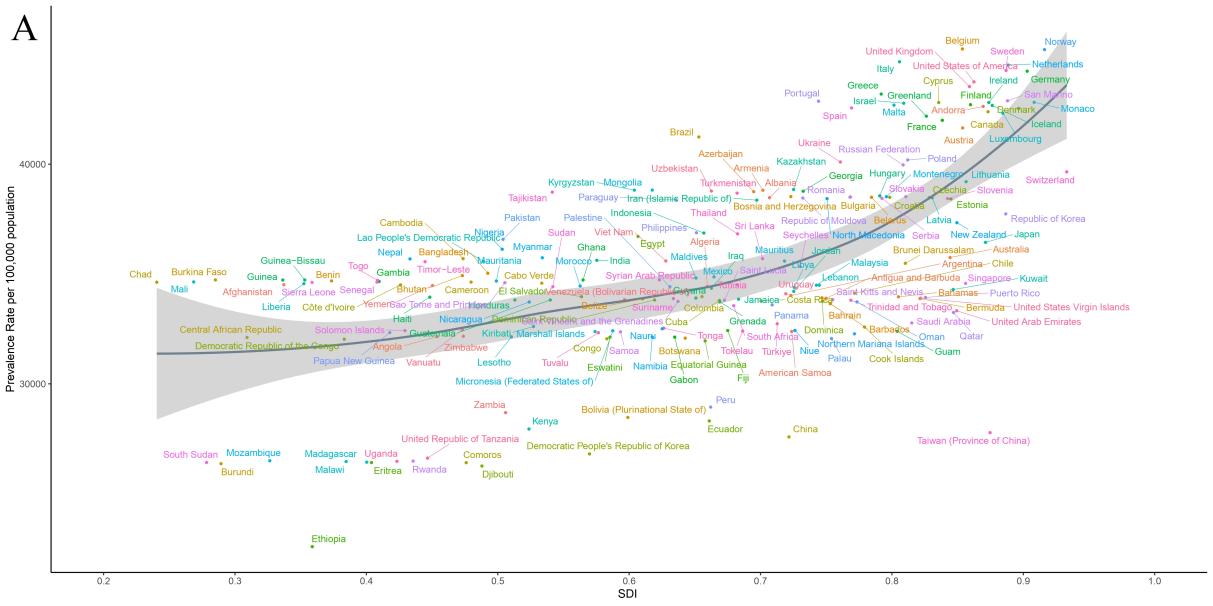


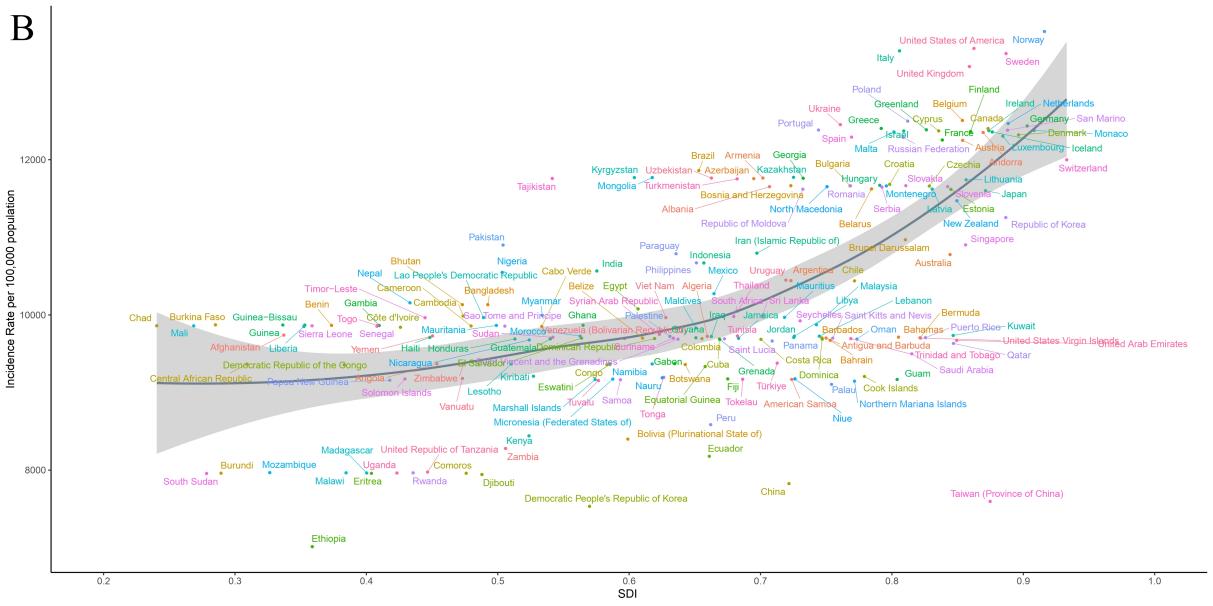


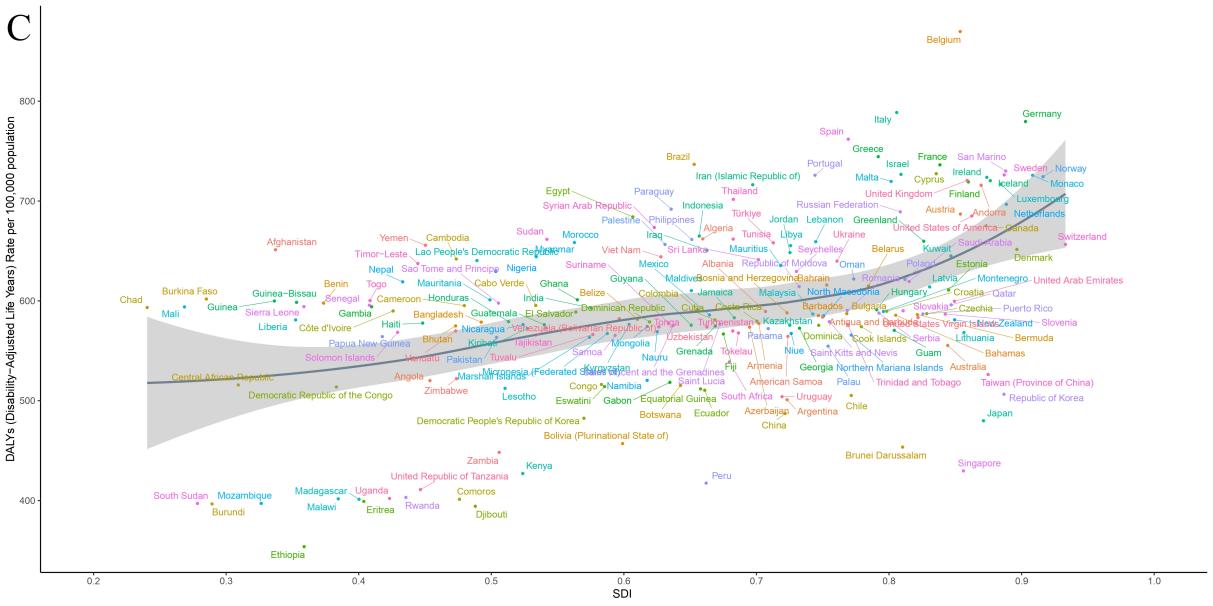


**Figure S8.** The associations between the SDI and headache disorder across 204 countries and territories. **A** Association between ASPR and SDI. **B** Association between ASIR and SDI. **C** Association between ASDR and SDI.

**Figure legend:** Panels A, B, and C display the relationship between SDI and age-standardized rates (per 100,000 population) for prevalence, incidence, and DALYs, respectively. Each country is labeled with its name, and the colors represent different regions. The shaded areas represent the 95% confidence intervals, and the blue line represents the best fit to the data. The data highlight the regional disparities and the varying association between SDI and health burden indicators across different regions of the world.

Abbreviations: ASPR, Age-Standardized Prevalence Rate; ASIR, Age-Standardized Incidence Rate; ASDR, Age-Standardized Disability-Adjusted Life Years; SDI, Socio-demographic Index; DALYs, Disability-Adjusted Life Years


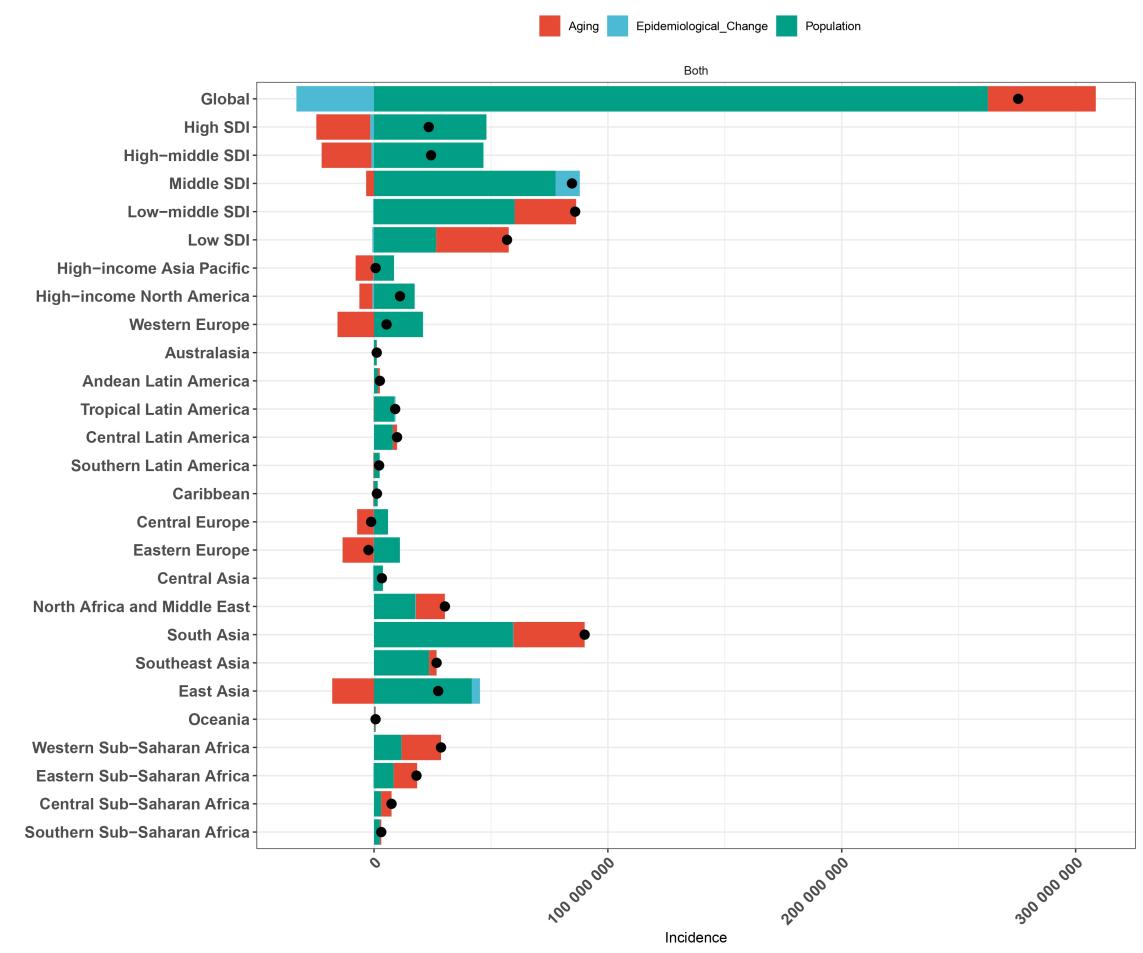


**Figure S9.** Decomposition analysis of headache disorder change in incidence by SDI and 21 GBD regions, 1990 to 2021

**Figure legend:** Decomposition of the change in the incidence of headache disorders from 1990 to 2021 by regions, showing contributions from aging, epidemiological change, and population growth. Red bars represent the contribution of aging, blue bars indicate epidemiological change, and green bars show population growth. The size of each bar corresponds to the respective regional contribution. The black dots represent the overall trend in disease burden change for each region.

**Abbreviations:** SDI, Sociodemographic Index; GBD, Global burden of disease


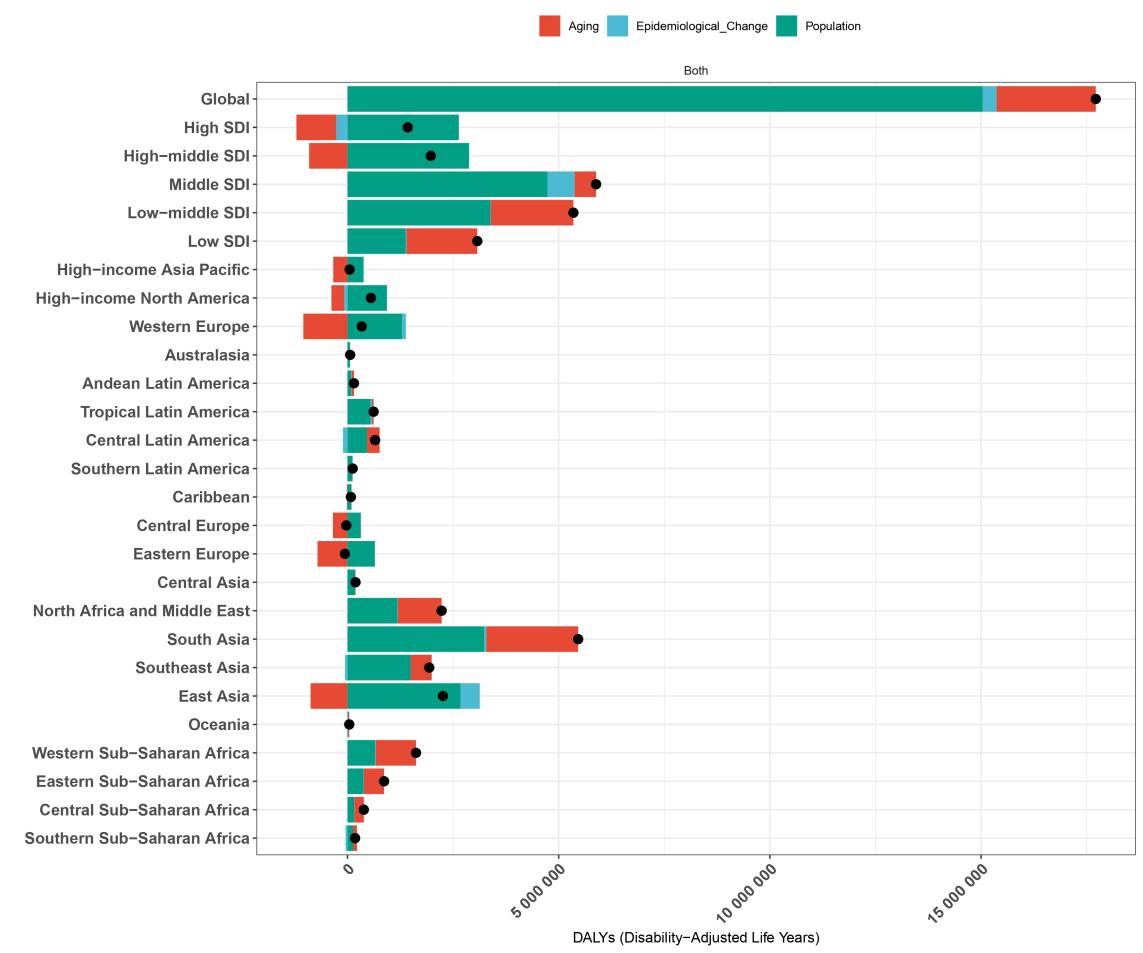


**Figure S10.** Decomposition analysis of headache disorder change in DALYs by SDI and 21 GBD regions, 1990 to 2021

**Figure legend:** Decomposition of the change in the DALYs of headache disorders from 1990 to 2021 by regions, showing contributions from aging, epidemiological change, and population growth. Red bars represent the contribution of aging, blue bars indicate epidemiological change, and green bars show population growth. The size of each bar corresponds to the respective regional contribution. The black dots represent the overall trend in disease burden change for each region.

**Abbreviations:** SDI, Sociodemographic Index; GBD, Global burden of disease; DALYs, Disability-Adjusted Life Years


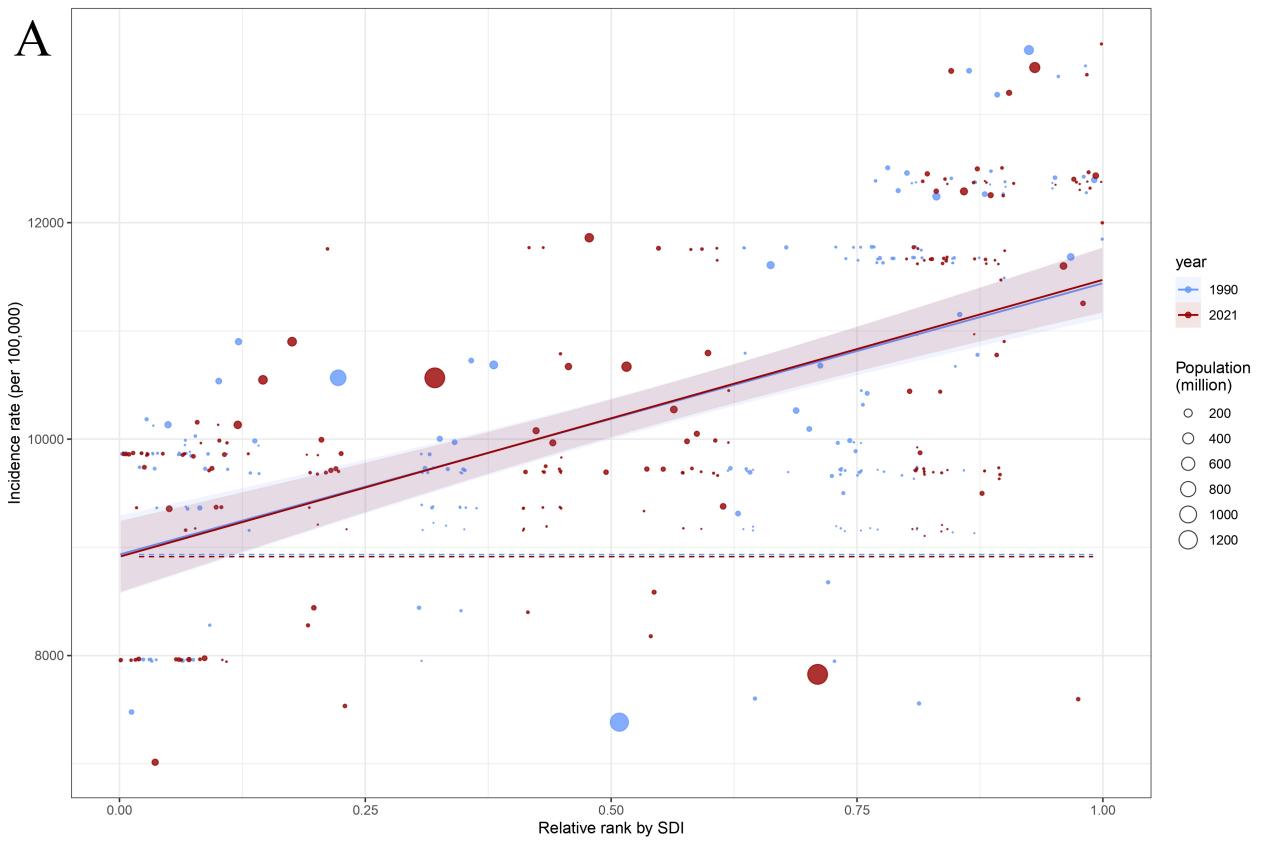

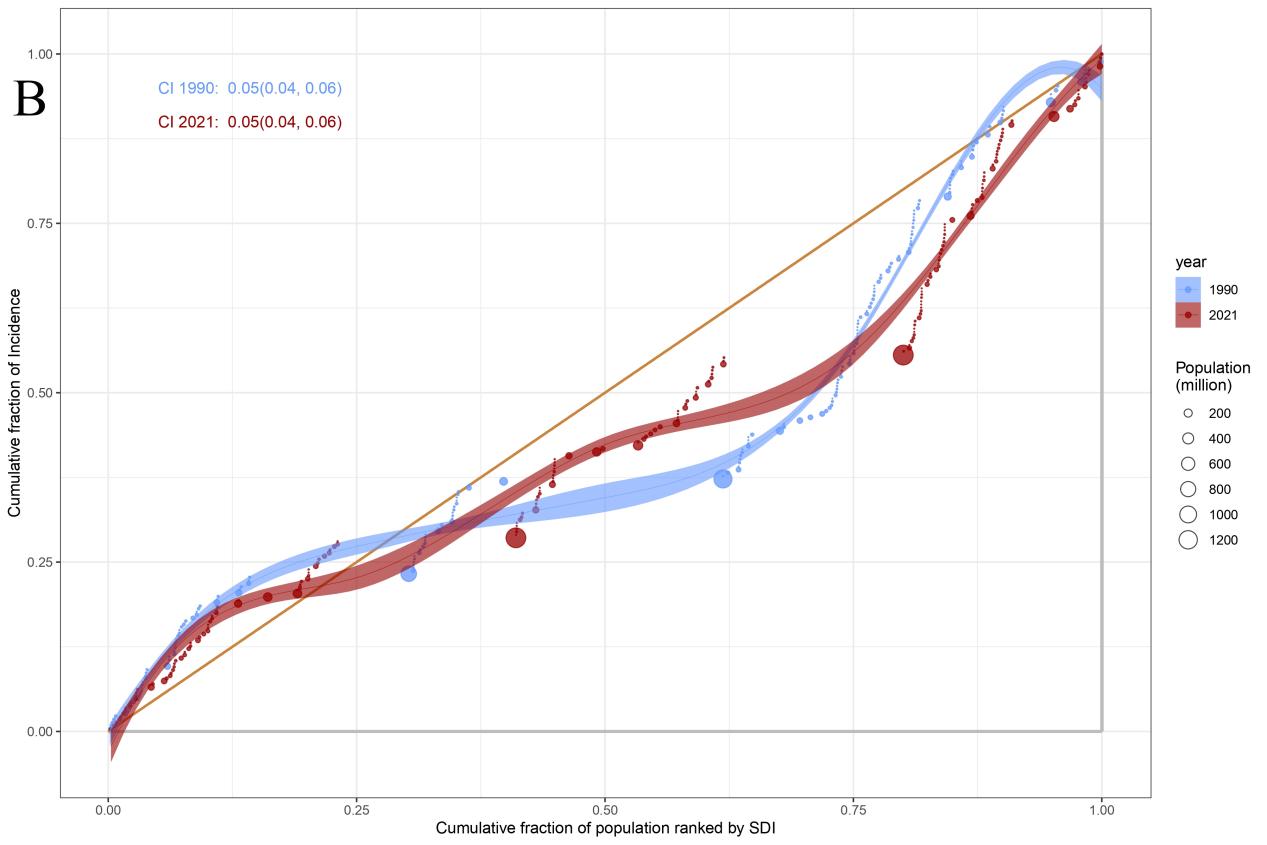


**Figure S11. A** Health inequality regression curves of incidence for headache disorders. **B** Concentration curves of incidence for headache disorders.

**Figure legend:**

(A) The relationship between ASIR and relative rank by SDI for headache disorders across 204 countries in 1990 and 2021. Points represent countries with varying population sizes, denoted by the size of the circle (population in millions). The blue dotted line represents the trend for 1990, and the red solid line shows the trend for 2021, along with shaded confidence intervals.

(B) The cumulative fraction of ASIR for headache disorders across 204 countries in 1990 and 2021. Points represent the cumulative fraction of incidence across different SDI categories, while the line reflects the cumulative trends in incidence rates. The CI values for 1990 and 2021 are shown as well.

**Abbreviations:** ASIR, Age-Standardized Incidence Rate; SDI, Sociodemographic Index; CI, concentration index


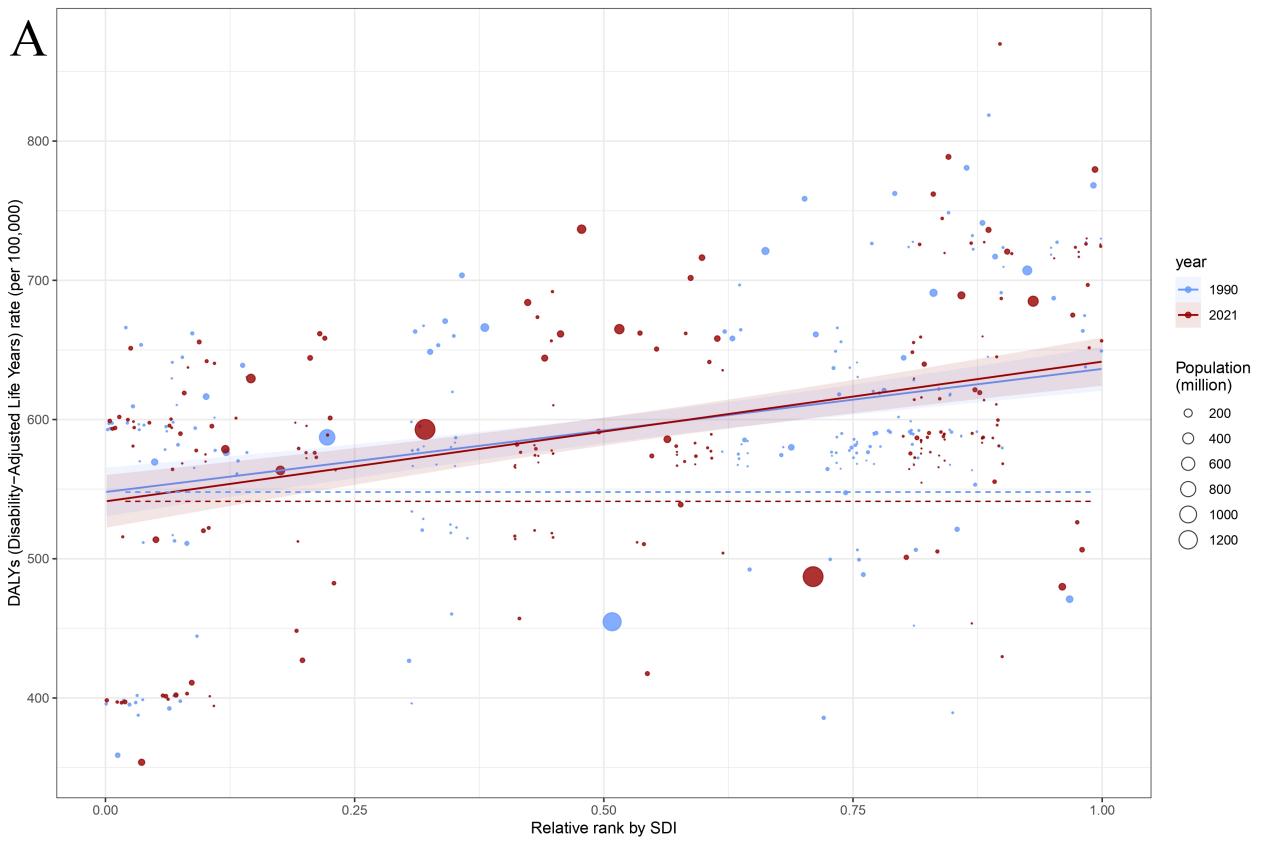


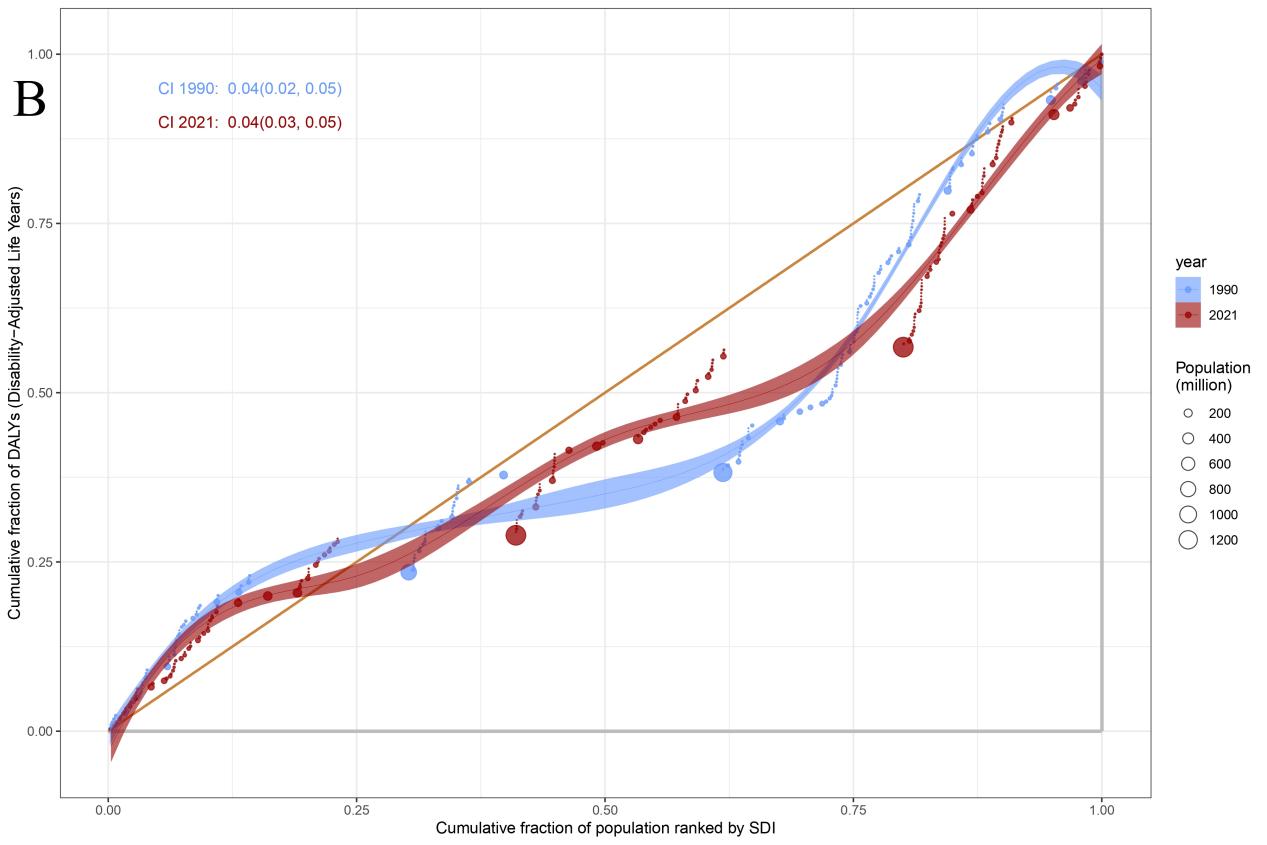


**Figure S12. A** Health inequality regression curves of DALYs for headache disorders. **B** Concentration curves of DALYs for headache disorders.

**Figure legend:**

(A) The relationship between ASDR and relative rank by SDI for headache disorders across 204 countries in 1990 and 2021. Points represent countries with varying population sizes, denoted by the size of the circle (population in millions). The blue dotted line represents the trend for 1990, and the red solid line shows the trend for 2021, along with shaded confidence intervals.

(B) The cumulative fraction of ASDR for headache disorders across 204 countries in 1990 and 2021. Points represent the cumulative fraction of DALYs across different SDI categories, while the line reflects the cumulative trends in incidence rates. The CI values for 1990 and 2021 are shown as well.

**Abbreviations:** ASDR, Age-Standardized DALYs Rate; SDI, Sociodemographic Index; CI, Concentration Index
